# Discriminator‐Guided Inverse Folding for Multi‐Property Protein Design

**DOI:** 10.1002/advs.75988

**Published:** 2026-06-09

**Authors:** Yuchuan Zheng, Chuyi Liu, Zhaoming Liu, Mao Su, Chenyu Tang, Xiang Zheng, Hao Zhang, Jingyuan Li

**Affiliations:** ^1^ Institute for Advanced Study in Physics Zhejiang University Hangzhou China; ^2^ School of Physics Zhejiang University Hangzhou China; ^3^ The Sixth Laboratory National Vaccine and Serum Institute (NVSI) Beijing China; ^4^ National Engineering Research Center for Novel Vaccines Beijing China; ^5^ Shanghai Artificial Intelligence Laboratory Shanghai China; ^6^ Laboratoire International Associé Centre National de la Recherche Scientifique et University of Illinois at Urbana‐Champaign Unité Mixte de Recherche n°7019 Université de Lorraine B.P. 70239 Vandœuvre‐lès‐Nancy cedex 54506 France

**Keywords:** discriminator‐guided optimization, inverse folding model, multi‐property optimization, structure‐based protein design

## Abstract

Designing proteins for real‐world applications requires the simultaneous satisfaction of multiple physicochemical properties. Structure‐based de novo protein design has become the prominent design paradigm, successfully creating numerous proteins. Property optimization is commonly introduced during the sequence generation stage of protein design, i.e., inverse folding. Existing methods primarily rely on fine‐tuning inverse folding models to design sequences with desired characteristics. However, multi‐property optimization through fine‐tuning demands datasets annotated with multiple properties—resources that remain extremely limited. Consequently, structure‐based protein design has not yet achieved joint optimization of multiple properties. Here, we present Discriminator‐Guided Inverse Folding (DGIF), a framework that guides the inverse folding model by adjusting its internal history states through an auxiliary discriminator module. The discriminator integrates multiple property predictors, each trained independently on a single‐property dataset, thereby enabling multi‐property optimization in the absence of datasets annotated with multiple properties. In addition to substantial improvements in key traits like thermostability and solubility, DGIF can generate protein sequences optimized for both properties simultaneously, with the designed proteins shifting markedly toward the Pareto front that represents optimal trade‐offs. Experimental results validate the effectiveness of DGIF for multi‐property protein design.

## Graphical abstract

Discriminator‐Guided Inverse Folding (DGIF) integrates multiple property predictors trained from single‐property datasets to guide protein sequence generation from a backbone structure. DGIF enables simultaneous improvement of thermostability and solubility without requiring multi‐property annotated datasets and generates designs that move toward the thermostability–solubility Pareto front.

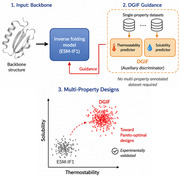

## 1 Introduction

Protein design aims to create new functional proteins to address the challenges in healthcare, agriculture, and sustainability [1, 2, 3]. Recent advances in structure‐based de novo design have enabled the generation of proteins that perform target functions under laboratory conditions [4, 5, 6, 7, 8, 9]. However, many designed proteins lack practical utility, primarily because real‐world applications demand additional protein properties [10, 11, 12, 13, 14]. For instance, industrial enzymes are typically employed at elevated temperatures to ensure catalytic efficiency [12, 15, 16]. Besides, their solubility is essential for the efficient production via recombinant expression systems [10, 17, 18]. Integrating property optimization into structure‐based design is therefore essential for translating the designed proteins into practical use.

Most protein traits are sensitive to sequence. Property optimization is commonly addressed during the sequence‐generation stage of protein design, i.e., inverse folding based on a given backbone structure [2, 19, 20, 21]. Recent efforts have attempted to generate protein sequence with desired properties by fine‐tuning inverse folding models using methods such as Supervised Fine‐Tuning (SFT) and Direct Preference Optimization (DPO) [22, 23, 24, 25]. However, it is still a challenge for these methods to achieve jointly optimization of multiple properties, as they rely on datasets annotated with multiple properties—resources that are extremely scarce. It should be noted that many properties, e.g., solubility and thermal stability, are inherently conflicting [26, 27, 28, 29]: mutations that enhance one trait tend to deteriorate another. This makes multi‐objective optimization particularly difficult. Given its importance for advancing the practical utility of protein design, there is a pressing need for strategies that can enable simultaneous optimization across multiple, and often competing, properties.

To address these challenges, we propose Discriminator‐Guided Inverse Folding (DGIF), a framework that guides the inverse folding model by adjusting its internal history states through an auxiliary discriminator module. The discriminator integrates multiple property predictors, each trained independently on a single‐property dataset, thereby enabling multi‐property optimization in the absence of datasets annotated with multiple properties. This guiding‐based framework does not require any further training or modification of the inverse folding model and can integrate into a given inverse folding model in a plug‐and‐play manner. We implement DGIF using the ESM‐IF1 model [21] as the base inverse folding architecture. In addition to substantial improvements in key traits like thermostability and solubility, DGIF can generate protein sequences optimized for both properties simultaneously, with the designed protein shifting markedly toward the Pareto front that represents optimal trade‐offs. In vitro experiments validated the effectiveness of DGIF, highlighting the ability of our discriminator‐guided strategy to integrate sequence generation with multi‐property control—a key step toward practical protein design.

## 2 Results

### 2.1 Inverse Folding Model

The goal of inverse folding is to generate sequences that fold into a desired backbone structure. Formally, this problem is represented as learning the conditional distribution *p*(*Y*|*X*). For a protein with *n* amino acids, *X* = (*x*_1_,…, *x*_i_,…, *x*_3n_) denotes the coordinates of each backbone atom N, Cα, and C, and *Y* = (*y*_1_,…, *y*_i_,…, *y*_n_) represents the generated sequence. Existing approach typically adopt an encoder‐decoder framework. In this framework, the encoder extracts the structural information from the backbone coordinate *X*, and the decoder predicts the conditional probability *p*(*Y* |*X*) in an autoregressive manner:

$$p\;\left(Y|X\right)=\prod_{t=1}^{n}p\left(y_{t}|y_{0},\text{…},y_{t-1};X\right)$$

Here, t denotes the current autoregression time step. At each step, the decoder takes as input the structural encoding *E_x_* produced by the encoder, along with a history state *H_t_* that encapsulates the context from the previously generated sequence *y*_1,_ *y*_2_,…,*y*_t._ It then produces an intermediate representation *o*_*t* + 1_. *o*_*t* + 1_ is subsequently projected by a linear output layer *W* into the probability distribution  *p*_*t* + 1_, from which the next amino acid *y*_*t* + 1_ can be sampled.

$$y_{t+1}∼\;\;p_{t+1}=Softmax\left(Wo_{t+1}H_{t},E_{x}\right)$$

By repeating this process, the inverse folding model can sequentially generate the entire amino acid sequence.

### 2.2 DGIF Framework

**FIGURE 1:**
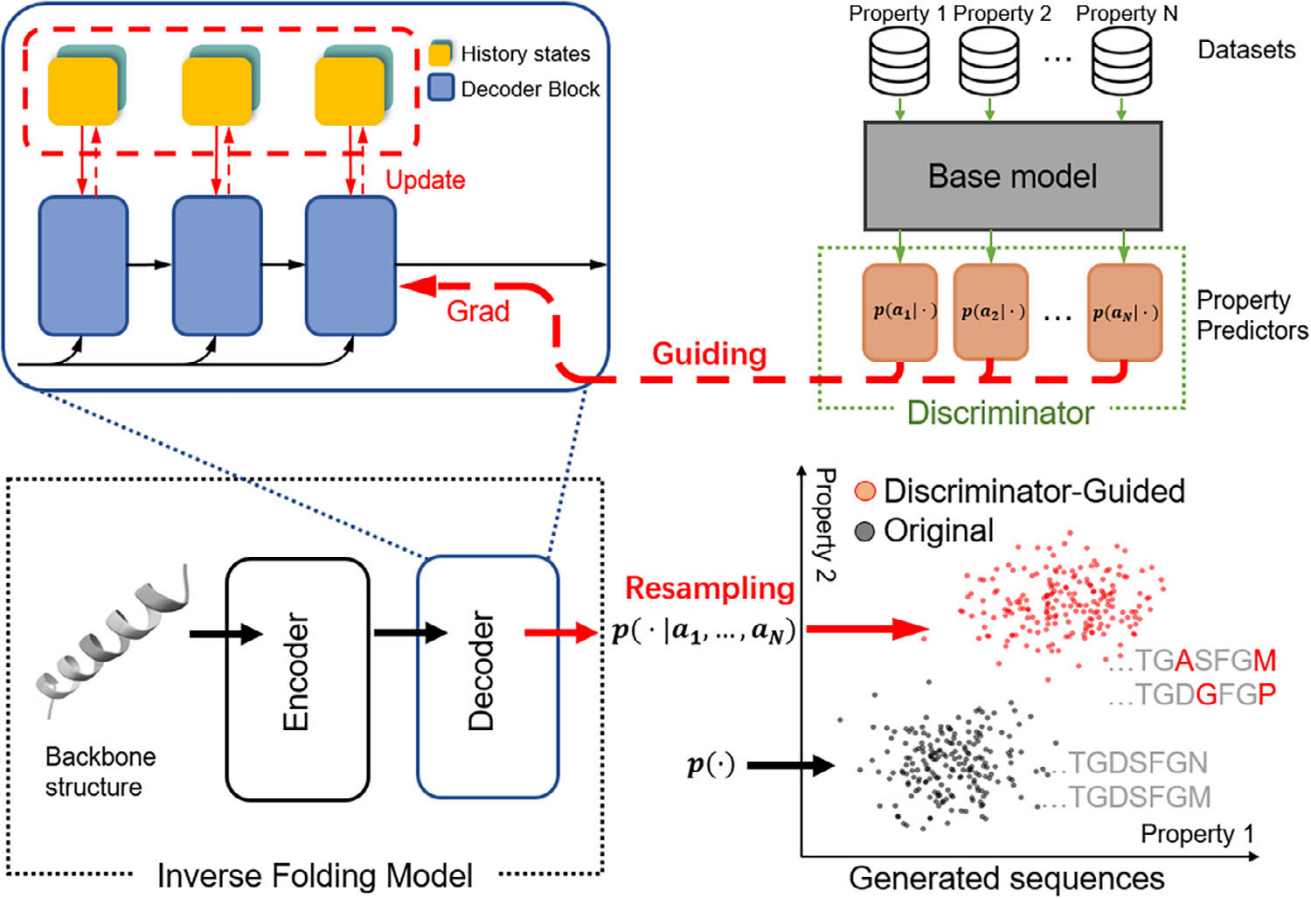
| Schematic of the DGIF framework. DGIF integrates an auxiliary discriminator into the inverse folding model to guide sequence generation toward desired traits. First, a forward pass is performed through the original inverse folding model. The discriminator then employs property predictors to assess the sequence's consistency with the target property (black arrows). Next, gradients from this feedback are backpropagated to update the decoder's history states, thereby increasing the probability of generating sequences that satisfy the desired traits (dashed red arrows). Finally, a new residue distribution is calculated based on the updated history states, and the next residue is re‐sampled (red arrows). This process is repeated at each time step, gradually shifting the sequence toward the target property. Notably, the discriminator can combine multiple predictors, each trained on single‐property datasets, thus enabling multi‐objective optimization without the need for multi‐property annotated data.

Inverse folding models are trained on naturally occurring protein structures, with the objective of maximizing the log‐likelihood of native sequences. As a result, these models are primarily optimized for sequence foldability and lack explicit guidance toward specific protein properties. To address this limitation, we introduce the **Discriminator‐Guided Inverse Folding (DGIF)** framework, which introduces an auxiliary discriminator to guide the sequence generation process (Figure 1). As previously described, at each time step *t*, the decoder predicts the next amino acid *y*_*t* + 1_ based on the history state *H_t_* and the structural encoding *E_x_*. In the DGIF framework, an external discriminator is applied to adjust *H_t_*. The discriminator leverages protein property predictors to evaluate whether the generated sequence satisfies the desired traits and propagates this signal backward to influence the generation trajectory. This modification can be viewed as a dynamic reinterpretation of the sequence history, with the goal of steering future predictions toward desired protein properties.

Specifically, in the case where the discriminator targets a desired property *a*, it employs a property predictor to estimate the probability that the generated sequence satisfies this property, conditioned on *H_t_* and *E_x_*, expressed as *p*(*a*|*o*_*t* + 1_(*H_t_*,*E_x_*)). Let Δ*H_t_* denote the adjustment applied to *H_t_*, such that generating with (*H_t_* + Δ*H_t_*) shifts the sequence distribution toward a higher likelihood of exhibiting the desired property. Initially, Δ*H_t_* is set to zero and iteratively updated using gradients from the predictor. The update rule is given by:

$$\Delta H_{t}\leftarrow \Delta H_{t}+\alpha \frac{\nabla_{\Delta H_{t}}\log p\left(a|o_{t}\left(H_{t}+\Delta H_{t},E_{x}\right)\right)}{∥\nabla_{\Delta H_{t}}\log p\left(a|o_{t}\left(H_{t}+\Delta H_{t},E_{x}\right)\right){∥}^{\gamma }}$$

$$\;{\tilde{H}}_{t}=H_{t}+\Delta H_{t}$$

Here, α is the step size, and γ is a normalization factor.

DGIF can further incorporate multiple property predictors to enable multi‐objective optimization. The discriminator integrates a set of predictors, *p*(*a_i_* |*o_t_* (*H_t_* + Δ*H_t_*,*E_x_*)), each associated with a weighting coefficient β_*i*_, to jointly update *H_t_*:

$$\Delta H_{t}\leftarrow \Delta H_{t}+\alpha \frac{\nabla_{\Delta H_{t}}\sum_{i}\beta_{i}\log p\left(a_{i}|o_{t}\left(H_{t}+\Delta H_{t},E_{x}\right)\right)}{∥\nabla_{\Delta H_{t}}\sum_{i}\beta_{i}\log p\left(a_{i}|o_{t}\left(H_{t}+\Delta H_{t},E_{x}\right)\right){∥}^{\gamma }}$$

$$\;{\tilde{H}}_{t}=H_{t}+\Delta H_{t}$$

By tuning the weighting coefficients β_*i*_, the relative influence of each property can be flexibly adjusted to balance trade‐offs between competing objectives.

After shifting the updated history state ${\tilde{H}}_{t}$, a forward pass through the decoder of the inverse folding model is performed to obtain the updated output ${\tilde{o}}_{t+1}$ and generate the next amino acid ${\tilde{y}}_{t+1}$:

$${\tilde{o}}_{t+1},\;H_{t+1}=Decoder\left(y_{t},\;{\tilde{H}}_{t},E_{x}\right)$$

$${\tilde{y}}_{t+1}∼\;\;{\tilde{p}}_{t+1}=Softmax\left(W{\tilde{o}}_{t+1}\right)$$

This process is repeated at each time step, enabling DGIF to guide sequence generation toward satisfying target properties, without altering any of the parameters of the inverse folding model.

In this work, we implement the DGIF framework on top of the ESM‐IF1 inverse folding model, resulting in the discriminator‐guided variants: **DG‐Thermo** (optimized for thermostability), **DG‐Sol** (optimized for solubility), and **DG‐Dual** (optimized simultaneously for both properties). These models demonstrate substantial improvements in target properties. DG‐Dual can successfully generate the sequences that exhibit enhancement in both thermostability and solubility.

### 2.3 Design Proteins With Enhanced Thermal Stability

Improving the thermal stability of proteins has long been an important objective [30, 31, 32, 33]. Most natural proteins function only under mild conditions [16], limiting their utility in many industry applications. On the other hand, proteins that remain active at elevated temperatures often exhibit increased catalytic efficiency and enhanced resistance to microbial contamination [12]. In this work, we introduce an external thermal stability predictor to guide the inverse folding model ESM‐IF1. The resulting discriminator‐guided model, DG‐Thermo, can effectively generate protein sequences with improved thermal stability.

We constructed a thermal stability predictor to quantify the effects of mutations on protein thermostability, using representations derived from the inverse folding model ESM‐IF1 (see Methods). Leveraging ESM‐IF1 as the encoder allows the predictor to refine its internal history states via backpropagation. The predictor was trained on the Megascale dataset [34], which contains over 700 000 experimentally measured mutation–stability pairs, and evaluated on two additional independent benchmarks (FireProt [35] and S669 [36]) to assess generalization. Compared with established thermostability prediction methods (FoldX [37], Rosetta [38], ThermoNet [39], and ThermoMPNN [40]), our predictor achieved substantially lower root‐mean‐square error (RMSE) than FoldX, Rosetta, and ThermoNet, and performed comparably to ThermoMPNN. These results demonstrate that our predictor provides accurate and generalizable stability estimation for downstream sequence design.

We further evaluated our thermostability predictor on the S669 benchmark and compared it with two ESM‐IF1‐based baselines: the original ESM‐IF1 and an ESM‐IF1 model fine‐tuned using Direct Preference Optimization (ESM‐IF1(DPO)). Our predictor achieved a Pearson correlation of 0.491, outperforming both ESM‐IF1 and ESM‐IF1(DPO) (Table S7). These results support the use of our thermostability predictor as a competitive guidance signal in DG‐Thermo.

**FIGURE 2:**
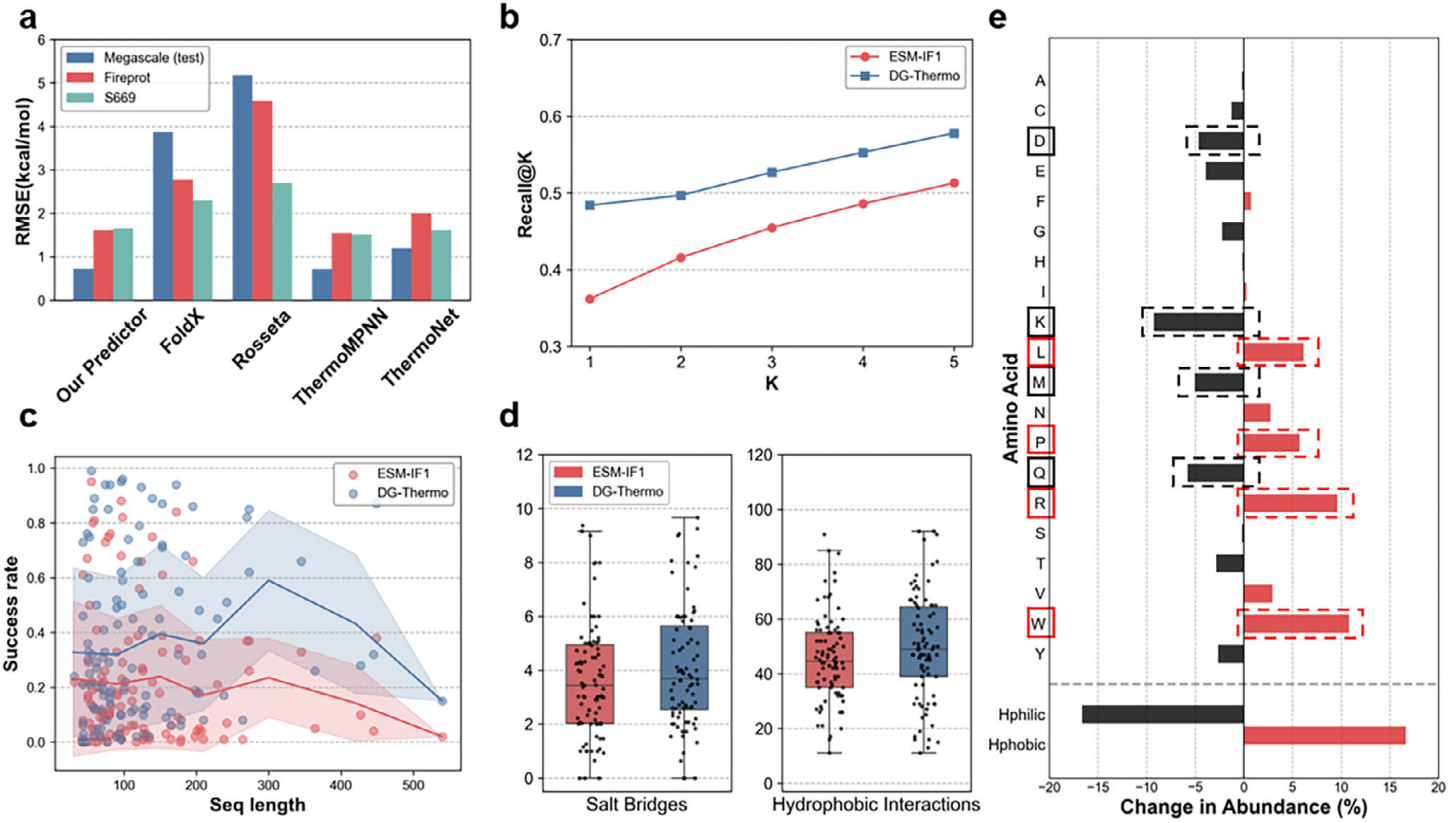
**|** Performance of **DG‐Thermo** in protein thermostability guidance. (a) Comparison of our thermostability predictor with predictors across different datasets. (b) Average top‐K recall on the Megascale test set comparing predictor‐guided **DG‐Thermo** with unguided ESM‐IF1. A higher top‐K recall indicates that mutations experimentally verified to improve thermostability are ranked with higher sampling probabilities. (c) Success rates of sequences designed by **DG‐Thermo** and ESM‐IF1. For each structure in the test set, 100 sequences were generated. A generated sequence is considered successful if it maintains foldability and exhibits improved thermostability. Solid lines and shaded areas indicate the mean and standard deviation of success rates, respectively. (d) Average number of salt bridges and hydrophobic interactions in designed proteins. (e) Changes in amino acid composition of proteins designed by **DG‐Thermo** with respect to those by ESM‐IF1. Amino acid types whose increased abundance is known to correlate with enhanced thermostability [12] are marked with red squares, the amino acids whose decreased abundance correlates with increased thermostability [12] are marked with black square.

Leveraging the thermal stability predictor, we developed a discriminator‐guided inverse folding model, DG‐Thermo. To evaluate its effectiveness in generating protein sequences with improved thermal stability, we compared its performance against the unguided ESM‐IF1. The evaluation was conducted on the Megascale test set, which comprises 86 proteins with experimentally measured thermal stability changes (ΔΔG) for all possible single‐residue mutations. Mutations with ΔΔG > 0—indicating increased thermal stability—were defined as positive samples, while the rest were considered negative samples. We assessed each model's ability to identify stabilizing mutations using the average top‐K recall. This metric is defined as the number of positive samples among the top K highest‐scoring mutations (as ranked by the model), divided by the total number of positive samples at each residue position. The metric reflects the model's capacity to prioritize beneficial mutations, thereby increasing their likelihood of being sampled. As shown in Figure 2b, DG‐Thermo consistently outperforms ESM‐IF1 across all K values, achieving higher average top‐K recall. These results demonstrate that incorporating the discriminator effectively steers sequence generation toward mutations that enhance thermal stability.

We further compared the sequence design success rates of DG‐Thermo and ESM‐IF1. For each backbone structure in the test set, 100 sequences were generated. A design was deemed successful if it met both of the following criteria: (1) the predicted ΔΔG from the thermal stability predictor exceeded 1.0 kcal/mol relative to the wild‐type sequence, and (2) the root‐mean‐square deviation (RMSD) between the predicted folded structure and the native structure was less than 2 Å. Figure 2c depicts the success rates of DG‐Thermo and ESM‐IF1 across proteins of varying lengths. Across all length intervals, DG‐Thermo consistently achieves significantly higher average success rates than ESM‐IF1. These results underscore the effectiveness of the DGIF framework in guiding the inverse folding model to design protein sequences with optimized thermal stability.

We observed that proteins designed by DG‐Thermo contained more salt bridges and hydrophobic interactions than those designed by ESM‐IF1 (Figure 2d). These structural features are widely recognized as key determinants of the higher thermostability of thermophilic proteins relative to their mesophilic counterparts [12, 41, 42]. In addition, sequence composition analyses comparing thermophilic and mesophilic proteins have shown that increased abundance of Leu (L), Pro (P), Arg (R), and Trp (W), along with decreased abundance of Asp (D), Lys (K), Met (M), and Gln (Q), are strongly correlated with enhanced protein thermostability [12]. We therefore compared the amino acid composition changes between proteins designed by DG‐Thermo and those generated by ESM‐IF1 (Figure 2e). The results revealed that DG‐Thermo closely reproduced these characteristic compositional shifts: L, P, R, and W showing the largest increases in abundance, and D, K, M, and Q showing the largest decreases. Notably, this information was not provided to the model as input, indicating that DG‐guided generation process inherently captures sequence and structure features associated with thermophilicity.

### 2.4 Evaluating the Thermal Stability of Designed Proteins via Molecular Dynamics (MD) Simulations

Molecular dynamics (MD) simulations have been widely used to investigate the thermal stability of proteins [12, 32, 43, 44]. To further evaluate the effectiveness of the DGIF framework, we employed MD simulations of proteins designed by DG‐Thermo as well as those generated by the unguided ESM‐IF1. As a representative example, we selected xylanase, an enzyme extensively used in industrial applications [45, 46].

**FIGURE 3:**
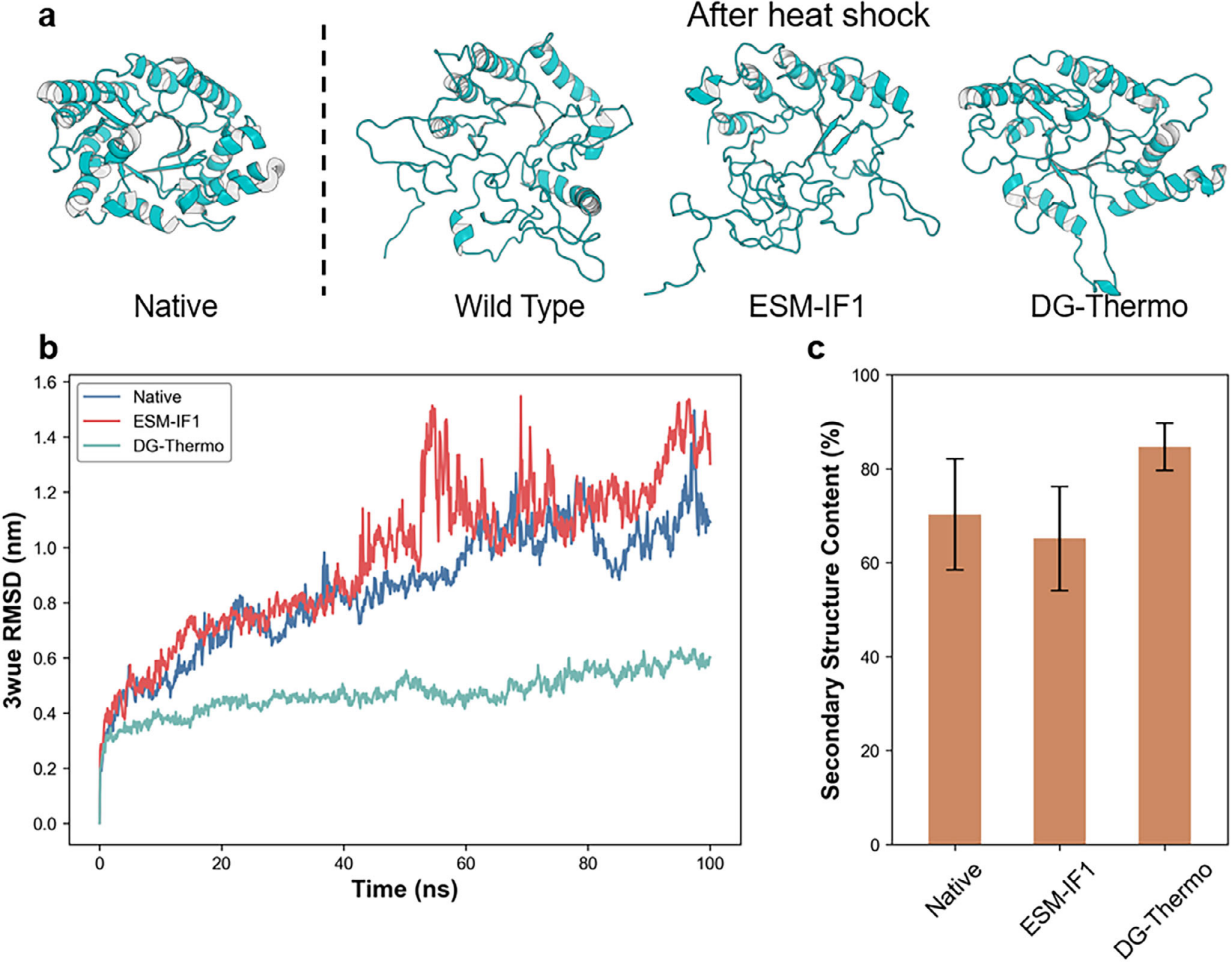
**|** Evaluating the thermal stability of designed proteins through molecular dynamics (MD) simulations. (a) Conformations of the wild‐type xylanase and its variants designed by DG‐Thermo and ESM‐IF1 after 100 ns simulations at an elevated temperature of 450 K. Left panel: native structure (PDB ID: 3WUE). Right panel (from left to right): final conformations of the proteins designed by DG‐Thermo, ESM‐IF1, and the wild type. (b) Root‐mean‐square deviation (RMSD) of designed protein and wild‐type protein with respect to their initial conformations. (c) Average secondary structure content in the designed proteins and wild‐type protein during 100‐ns simulations.

We performed 100 ns MD simulations at an elevated temperature of 450 K for the DG‐Thermo‐designed variants, the ESM‐IF1‐designed variant, and the wild‐type xylanase [45] (see Methods). Three DG‐Thermo‐designed variants were considered in this work. Following heat shock, all DG‐Thermo‐designed variants largely retained their initial structure, whereas both the ESM‐IF1‐designed variant and the wild type exhibited pronounced structural collapse (Figure 3a, Figure S1). Throughout the simulations, the DG‐Thermo‐designed variant maintained a lower and more convergent RMSD (all less than 5 Å), while the RMSD values of the ESM‐IF1‐designed variant and the wild type were substantially higher and diverged over time (Figure 3b). Secondary structure analysis further showed that all DG‐Thermo‐designed variants retain >83.28% of their secondary structure during the simulation, compared with 71.13% for the wild type and 65.91% for the ESM‐IF1‐designed variant. Collectively, these results indicate that the xylanase redesigned by DG‐Thermo exhibits markedly greater thermal stability than both the ESM‐IF1‐designed and native forms, thereby validating the effectiveness of the DGIF framework.

In addition, we randomly sampled ten protein scaffolds from the CATH4.3 dataset [47] for sequence redesign and evaluated the thermal stability of the DG‐Thermo‐designed variants, ESM‐IF1‐designed variants, and the wild type through MD simulations (Figure S2a–j). Across all cases, the DG‐Thermo‐designed variants consistently exhibited enhanced thermal stability.

### 2.5 Retrospective Analysis of Thermostability‐Directed Evolution Cases

We conducted a retrospective analysis of reported thermostability‐directed evolution studies to examine whether DG‐Thermo can prioritize experimentally retained mutations and variants. We selected two protein systems with well‐documented evolutionary trajectories and thermal‐stability measurements [48, 49]: subtilisin E (PDB 1SCJ) and Thermus maltogenic amylase (PDB 1SMA) (Table S3A,B). For each mutation retained in the reported trajectories, we enumerated all 19 possible non‐wild‐type substitutions at the same site and ranked them by the backbone‐conditioned sequence log‐likelihoods assigned by DG‐Thermo and unguided ESM‐IF1. In the 1SCJ case, DG‐Thermo ranked 5 of the 8 retained mutations first. In the 1SMA case, DG‐Thermo ranked 3 of the 7 retained mutations first and 4 within the top 5. By contrast, unguided ESM‐IF1 generally assigned lower ranks to these mutations (Table S6A,B). These results suggest that the thermostability discriminator provides useful additional information for prioritizing candidate mutations.

We further calculated the log‐likelihoods of the reported variants along the two directed‐evolution trajectories. In both systems, DG‐Thermo assigned substantially higher log‐likelihoods to the final successful multi‐mutant variants than to the corresponding wild‐type sequences, whereas the log‐likelihood changes assigned by unguided ESM‐IF1 remained close to zero (Table S5A,B and Figure S7). Notably, the log‐likelihood gains of some multi‐mutant combinations were not equal to the summed gains of their component single mutations, suggesting that DG‐Thermo evaluates combinatorial variants in a context‐dependent manner rather than as a simple accumulation of independent single‐mutation effects (Tables S4A,B and S5A,B). Overall, these retrospective analyses suggest that DG‐Thermo can prioritize experimentally retained mutations among alternative substitutions at the corresponding mutated sites and assign higher log‐likelihoods to reported successful multi‐mutant variants. These findings indicate that DGIF may provide useful scoring information for directed‐evolution‐assisted protein optimization.

### 2.6 Design Proteins With Enhanced Solubility

Natural selection has tuned protein solubility to meet physiological requirements, which can pose challenges when proteins are used at high concentrations in recombinant applications [10, 50]. Improving protein solubility is therefore an important design objective. Here, we integrated a solubility predictor into ESM‐IF1 using the DGIF framework, yielding in the model DG‐Sol for designing proteins with enhanced solubility.

The solubility predictor employs a network architecture analogous to that of the thermostability predictor (see Methods). It was trained on a dataset compiled by Khurana et al. [51], comprising 28 972 soluble and 40 448 insoluble protein sequences, with a 9:1 split between training and validation sets. An independent test set curated by Chang et al. [50], containing 1000 soluble and 1001 insoluble sequences, was used for evaluation. We benchmarked our predictor against three widely used solubility prediction methods—SoluProt [52], DeepSol [51], and PROSSO II [53]. As shown in Table 1, our method achieved higher accuracy (0.72) and Matthews correlation coefficient (MCC) (0.42) than both SoluProt (0.68 and 0.38) and PROSSO II (0.64 and 0.34), while performing comparably to DeepSol (0.73 and 0.46). These results demonstrate that our solubility predictor provides accurate and generalizable solubility estimation, ensuring reliable property evaluation for downstream sequence design within the DGIF framework.

**TABLE 1:** Performance of our solubility predictor and existing approaches.

Method	Accuracy	MCC	Precision	Recall
Our predictor	0.72	0.42	0.74	0.62
SoluProt	0.68	0.38	0.70	0.72
DeepSol	0.73	0.46	0.75	0.69
PROSSO II	0.64	0.34	0.67	0.69

**FIGURE 4:**
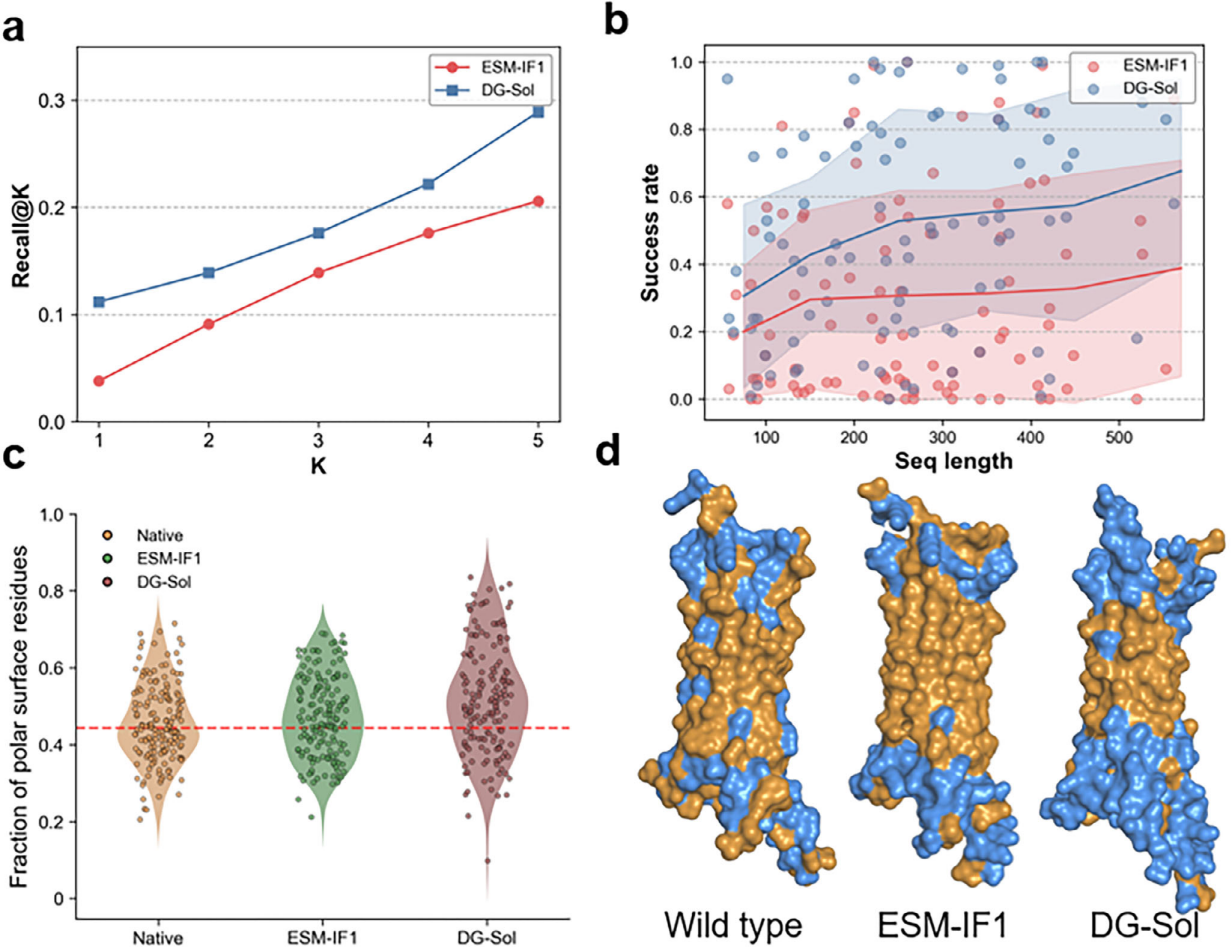
| Performance of DG‐Sol in protein solubility guidance. (a) Average top‐K recall of predictor‐guided DG‐Sol versus unguided ESM‐IF1. Higher top‐K recall indicates that mutations experimentally validated to improve solubility are assigned with higher sampling probabilities. (b) Success rates of sequences designed by DG‐Sol and ESM‐IF1. For each structure in the test set, 100 sequences were generated. A generated sequence is considered successful if it maintains foldability and exhibits improved solubility. Solid lines and shaded areas indicate the mean and standard deviation of success rates, respectively. (c) Proportion of surface polar residues of model‐designed variants and native proteins. The red line indicates the median value of native sequences. (d) Distribution of polar residues (blue) and nonpolar residues (yellow) on the surface of claudin‐15. From left to right: native protein, variant designed by ESM‐IF1, and variant designed by DG‐Sol.

We leveraged the DGIF framework to integrate the solubility predictor into the original ESM‐IF1, yielding a solubility predictor–guided inverse folding model, DG‐Sol. To evaluate its ability to generate protein sequences with improved solubility, we compared its performance with the unguided ESM‐IF1. Specifically, we first assessed each model's capacity to identify solubility‐enhancing mutations using the SoluProtMut^DB^ dataset [54], which contains proteins with experimentally measured solubility changes for all possible single‐residue substitutions. Mutations that improved solubility were denoted as positive samples, and all others as negative samples. We then calculated the average top‐K recall for both models. As shown in Figure 4a, DG‐Sol consistently outperformed ESM‐IF1 across all K values, indicating that the discriminator effectively guides the model to identify and preferentially sample residues conducive for solubility improvement.

We further compared the sequence design success rates of DG‐Sol and ESM‐IF1. For each backbone structure in the test set, 100 sequences were generated. A design was considered successful if it satisfied both of the following criteria: (1) the predicted solubility of the designed sequence exceeded that of the wild type, and (2) the root‐mean‐square deviation (RMSD) between the predicted folded structure and the native structure was less than 2 Å. As shown in Figure 4b, DG‐Sol consistently achieved a substantially higher average success rate than ESM‐IF1 across all protein length ranges. These results underscore the effectiveness of the DGIF framework in markedly enhancing the model's ability to design protein sequences with improved solubility.

We evaluated the performance of DG‐Sol against ESM‐IF1 in designing high‐solubility variants using the membrane protein dataset curated by Koehler Leman et al. [55], Membrane proteins are typically poorly soluble, and enhancing their solubility is essential for industrial applications as well as the development of therapies [4]. In this work, we compared the proportion of surface polar residues in the designed proteins, as surface polarity is a key determinant of protein solubility [4]. As shown in Figure 4c,d, proteins designed by DG‐Sol contained a significantly higher proportion of surface polar residues than those designed by ESM‐IF1, underscoring the effectiveness of the DGIF framework in guiding the model to generate proteins with improved solubility.

### 2.7 Design Proteins With Simultaneously Enhanced Thermal Stability and Solubility

Proteins often need to exhibit multiple physicochemical properties to meet the demands of practical applications. For instance, industrial enzymes must combine high thermal stability with good solubility to maintain functional performance under harsh conditions and enable efficient recombinant production [10, 12]. However, numerous studies have documented a pronounced trade‐off between these properties: mutations that improve one trait often compromise the other [10, 26, 28]. Consequently, achieving concurrent optimization of both remains a significant challenge in protein engineering. In this work, we demonstrate that the DGIF framework can effectively address this trade‐off. By integrating thermal stability and solubility predictors directly into the ESM‐IF1 sequence generation process, we created DG‐Dual—a model capable of designing novel protein sequences that exhibit both enhanced thermal stability and high solubility.

**FIGURE 5:**
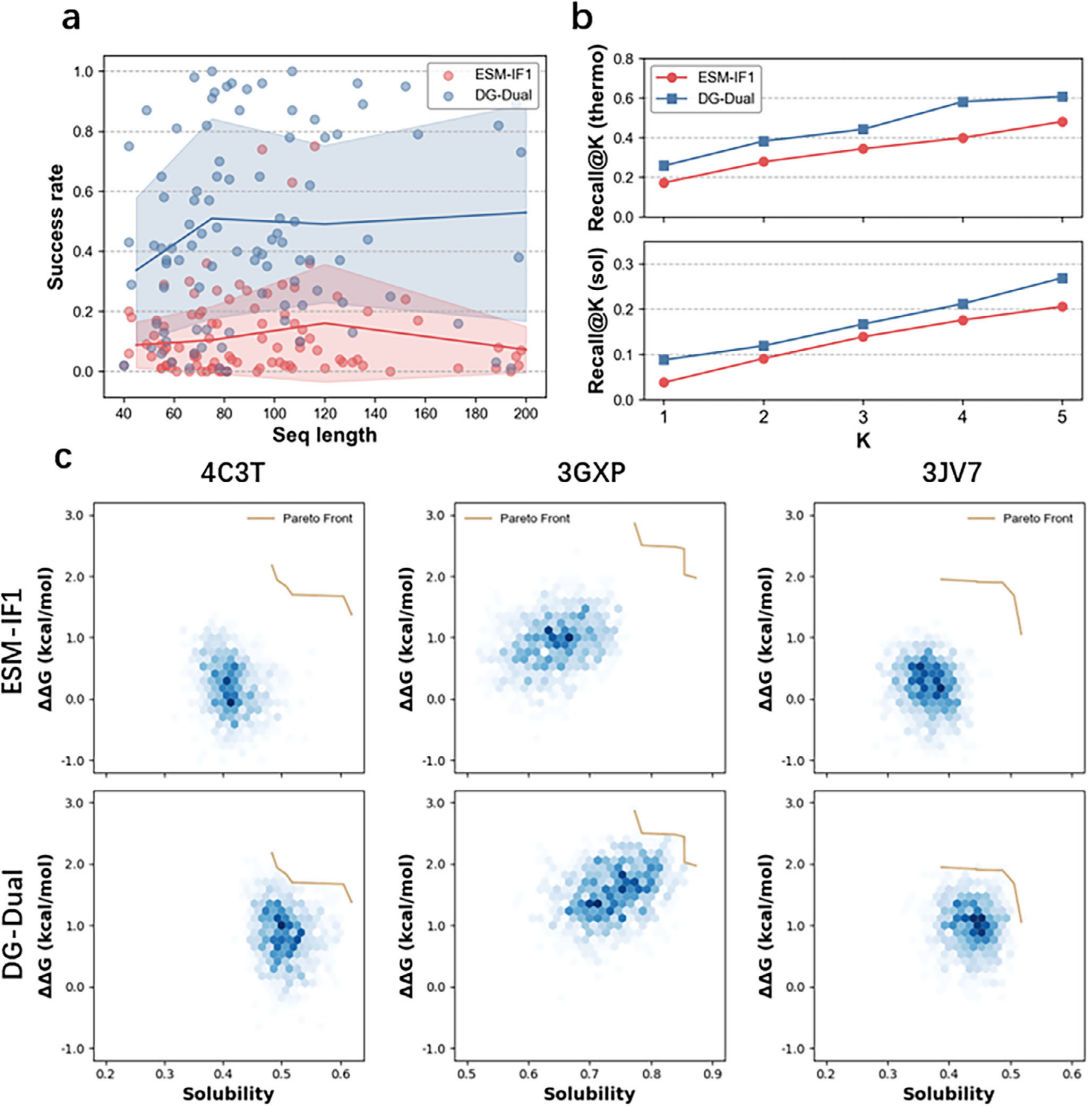
| Performance of DG‐Dual in designing proteins with both enhanced thermal stability and solubility. (a) Success rates of sequences designed by DG‐Dual and ESM‐IF1 under multiple design criteria. A design was considered successful if it maintained foldability while showing simultaneous improvements in both thermal stability and solubility. (b) Average top‐K recall of the models on experimental datasets for thermal stability and solubility. (c), Predicted ΔΔG values and solubility scores for proteins redesigned by DG‐Dual and ESM‐IF1, based on 1000 sequences generated by each model.

We compared the success rates of DG‐Dual and ESM‐IF1 in designing sequences optimized concurrently for thermal stability and solubility. To conduct the evaluation, 100 proteins were randomly sampled from the CATH4.3 dataset [47], and for each backbone structure, both models generated 100 sequences. A design was considered successful if it met all three criteria: (1) the predicted ΔΔG from the thermal stability predictor exceeded 1.0 kcal/mol relative to the wild‐type, (2) the predicted solubility was higher than the wild type, and (3) the root‐mean‐square deviation (RMSD) between the predicted folded and native structures was less than 2 Å. As shown in Figure 5a, DG‐Dual consistently achieved high success rates across all protein length ranges, whereas ESM‐IF1 performed poorly. These results indicate that the guidance of the discriminator imparts robust multi‐objective optimization capabilities, enabling the design of sequences that concurrently improve thermal stability and solubility.

We further evaluated the average top‐K recall of DG‐Dual and ESM‐IF1. Thermal stability and solubility metrics were computed using the Megascale test set and SoluProtMut^DB^ dataset, respectively, following the methodology described above. As shown in Figure 5b, DG‐Dual consistently outperformed ESM‐IF1 across all K values on both datasets. Remarkably, even when both thermal stability and solubility predictors were applied simultaneously, its average top‐K recall remained virtually identical to that achieved when optimizing a single property. This demonstrates that the DGIF framework can preserve the model's ability to optimize individual properties while enabling effective and balanced multi‐objective optimization.

Subsequently, we used DG‐Dual and ESM‐IF1 to redesign proteins and evaluated the thermal stability and solubility of the resulting variants. As shown in Figure 5c and Figure S3, compared with ESM‐IF1, proteins generated by DG‐Dual shift markedly toward the Pareto front, which represents optimal trade‐offs between stability and solubility. This result clearly demonstrates that our framework effectively steers ESM‐IF1 toward dual‐property optimization through coordinated guidance from two property‐specific predictors, achieving simultaneous improvements in both thermal stability and solubility without requiring datasets annotated with multiple properties.

### 2.8 Experimental Validation of DG‐Dual

**FIGURE 6:**
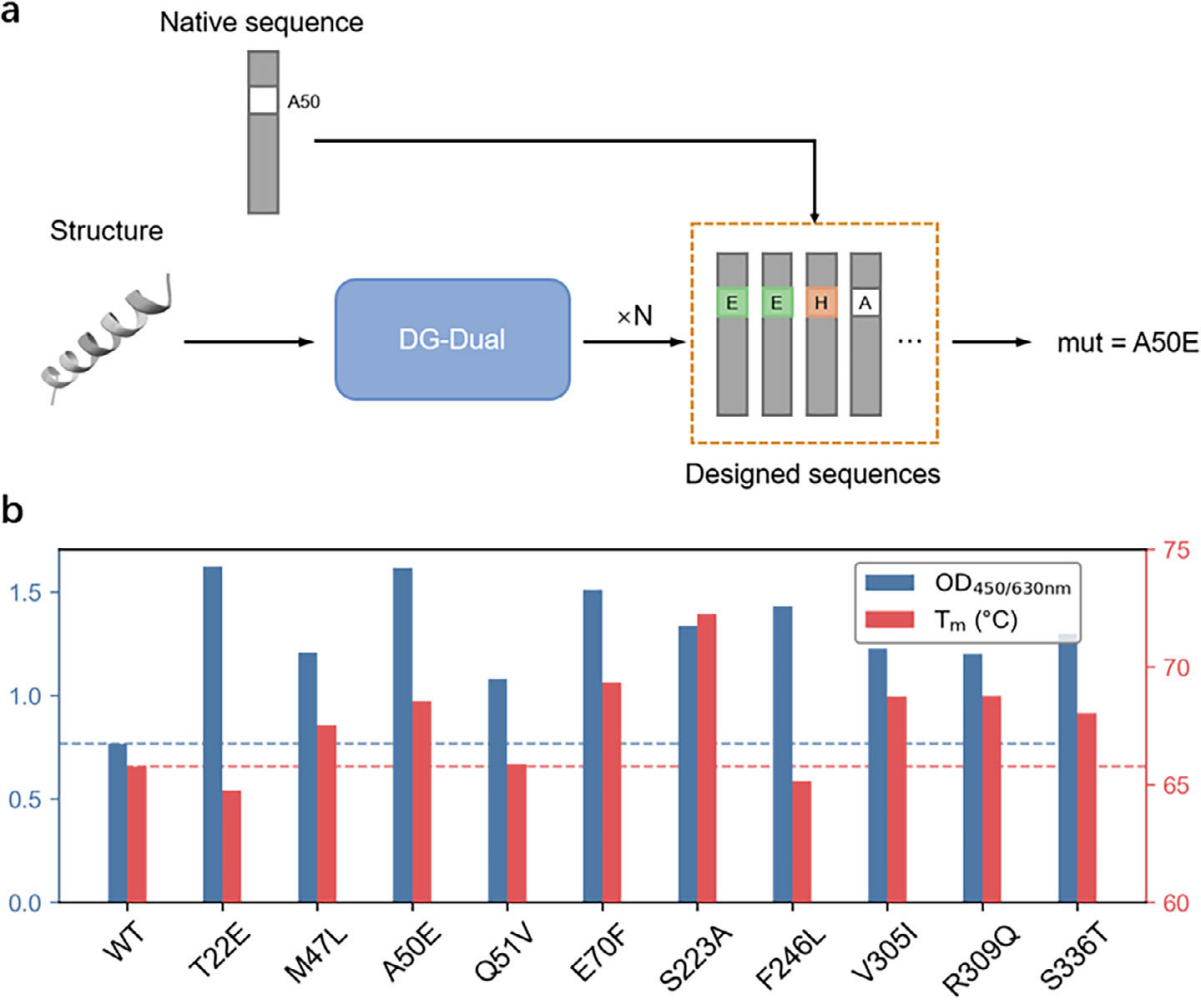
**|** Experimental validation of DG‐Dual for optimizing Rhodococcus ruber alcohol dehydrogenase (RrADH). (a) Workflow of DG‐Dual guided sequence redesign. DG‐Dual generated 10,000 structurally compatible sequences, and the amino acid occurrence frequency at each residue position was calculated. Mutations showing higher occurrence frequencies than the corresponding wild‐type residues were identified as key point mutations. (b) Experimentally measured solubility, represented by the value of optical density OD_450/630 nm_ in ELISA, and thermostability, represented by melting temperature (Tm), of the DG‐Dual designed RrADH variants and the wild type.

To assess the capability of DG‐Dual to simultaneously optimize multiple physicochemical properties of proteins, we used Rhodococcus ruber alcohol dehydrogenase (RrADH) as a case study, and the optimization effects were validated by wet‐lab experiments. RrADH is an organic solvent–tolerant oxidoreductase widely used in asymmetric synthesis [56, 57]. However, it is characterized by low solubility during heterologous expression, which hampers efficient recombinant production and limits its industrial applications [56, 58]. We applied DG‐Dual to identify key point mutations in RrADH that could concurrently enhance solubility and thermostability (Figure 6a). Specifically, DG‐Dual performed sequence redesigns based on the backbone structure of RrADH, and candidate mutations were subsequently identified by comparing the predicted amino acid probabilities at each residue position with those of the native sequence. In total, ten single‐point mutations were selected for experimental validation (see Methods).

We expressed both the wild‐type and DG‐Dual designed variants of RrADH in *Escherichia coli* (*E. coli*) and evaluated their solubility and thermostability (see Methods). The solubility of proteins was determined using enzyme‐linked immunosorbent assay (ELISA) method, and thermostability was represented by the melting temperature (Tm) measured using the Uncle platform (Unchained Labs, USA). As shown in Figure 6b and Table S1, all DG‐Dual designed variants showed enhanced solubility relative to the wild type, with nine variants exhibiting more than a 50% increase of OD_450/630 nm_ values in ELISA, including four that exceeded an 80% improvement. Moreover, the DG‐Dual designed mutations also enhanced the thermostability of RrADH: eight out of ten variants exhibited higher melting temperatures (Tm) than the wild type (Figure 6b and Figure S6). Notably, the A50E mutation resulted in a twofold increase in OD_450/630 nm_ values accompanied by a 2.79 °C increase in Tm, while the S223A mutation improved Tm by 6.47 °C with a concurrent 74% increase in OD_450/630 nm_ values. Collectively, these results demonstrate that DG‐Dual enables effective and simultaneous optimization of multiple physicochemical properties.

## 3 Discussion

In this study, we present Discriminator‐Guided Inverse Folding (DGIF), a framework to achieve multi‐property optimization in structure‐based protein design. DGIF steers the sequence generation of the inverse folding model through an auxiliary discriminator module. The discriminator integrates multiple property predictors that were separately trained on single‐property datasets, thereby enabling multi‐property optimization in the absence of datasets annotated with multiple properties. We systematically evaluated the DGIF framework on two inherently conflicting properties—thermostability and solubility—and found that it not only achieves substantial improvements in each property individually but also balances their trade‐off. Compared with the unguided ESM‐IF1, DGIF‐guided designs shift markedly toward the Pareto front, approaching optimal trade‐offs between stability and solubility. Importantly, in vitro experiments validated that DGIF can simultaneously optimize both thermostability and solubility of the target protein.

The DGIF framework intervenes exclusively in the history states during sequence generation, thereby preserving both the parameters and architecture of the base model. To ensure that property optimization does not compromise structural feasibility, we introduce a Kullback–Leibler (KL) divergence constraint that limits deviations of the regulated sampling distribution from the original model. Analysis of the relationship between protein properties and foldability (Figure S5) shows that DGIF can substantially enhance target properties while preserving structural integrity. Moreover, we observed a difference in the convergence rates of the losses for thermostability and solubility (Table S2), indicating an imbalance between these two objectives that complicates their joint optimization. To address this imbalance, we assign different weighting coefficients to each property through hyperparameter search. This weighting strategy likely contributes to the effectiveness of DGIF in multi‐property optimization.

DGIF provides a flexible strategy for multi‐property protein design without requiring jointly annotated multi‐property datasets. However, its property‐control capability still depends on the reliability of the single‐property predictors used for guidance, which is constrained by the availability of labeled data for the corresponding properties. Although single‐property annotations are generally easier to obtain than jointly annotated multi‐property datasets, data availability varies substantially across protein properties. For properties with relatively abundant public data, such as protein abundance and aggregation propensity [59, 60], DGIF can be extended by training property predictors from existing datasets. For more specialized properties, such as catalytic activity and substrate specificity, public datasets are often smaller and more system‐dependent. In these cases, DGIF may be more suitable for system‐specific optimization and may require additional experimental data to obtain reliable guidance signals.

One possible way to reduce the dependence on experimentally labeled datasets is to incorporate physics‐based or empirical scoring signals into DGIF. For example, scoring functions such as Rosetta [61] and FoldX [62] have been used to evaluate protein properties such as binding affinity and folding stability without requiring additional experimentally labeled training data [61, 63]. These scoring signals could be integrated into the discriminator‐guided generation process as property‐related guidance signals. With the continued development of more advanced structure‐conditioned generative models and the accumulation of relevant data, DGIF may be further extended to design tasks involving protein–small molecule complexes, protein–protein complexes, or protein–RNA complexes.

## 4 Method

### 4.1 Esm‐If1

Inverse folding aims to generate a sequence Y that reliably folds into a target backbone structure X. ESM‐IF1 [21], a Transformer‐based autoregressive model, addresses this task by first encoding the structural features of X with its encoder, and then autoregressively modeling the conditional probability distribution of the output sequence through the decoder:

$$p\;\left(Y|X\right)=\prod_{t=1}^{n}p\left(y_{t}|y_{t-1},\text{…},y_{1};X\right)$$

where Y is the designed sequence, t is the current autoregressive step, and n is the sequence length. To accelerate inference, the model maintains a key–value (KV) cache that stores contextual information from previous steps. The history state is defined as: $\;H_{t}=\left[\left(K_{0∼t}^{\left(1\right)},V_{0∼t}^{\left(1\right)}\right),\text{…},\left(K_{0∼t}^{\left(l\right)},V_{0∼t}^{\left(l\right)}\right)\right]$where $\left(K_{0∼t}^{\left(i\right)},V_{0∼t}^{\left(i\right)}\right)$ denotes the cached keys and values of the i‐th Transformer block from step 0 to t. At each step, the decoder consumes the sampled amino acid *y_t_* together with the stored *H_t_*, producing the next hidden representation and updating the cache:

$$o_{t+1},H_{t+1}=Decoder\left(y_{t},H_{t},E_{x}\right)$$

where *E_x_* is the structural encoding of *X* obtained from the encoder, and *o_t+1_* is the decoder output. A linear projection layer *W* maps *o_t+1_* into a probability distribution, from which the next amino acid is sampled:

$$y_{t+1}∼\;\;p_{t+1}=Softmax\left(Wo_{t+1}\right)$$

Thus, the prediction of *y_t+1_* depends on the history state *H_t_* and the fixed structure encoding *E_x_*.

### 4.2 Discriminator‐Guided Inverse Folding

Discriminator‐Guided Inverse Folding (DGIF) guides the autoregressive generation of ESM‐IF1 by introducing an auxiliary discriminator that modifies the history state *H_t_*. This modification serves as a dynamic reinterpretation of the sequence history, steering subsequent predictions toward desired properties [64].

Specifically, the discriminator uses a protein property predictor to estimate the probability that the generated sequence satisfies a property *a*, conditioned on (*Ht*, *E_x_*):

$$p(a|o_{t}\left(H_{t},E_{x}\right))$$

DGIF uses the discriminator to backpropagate and update the history state *H_t_* toward increasing *p*(*a*|*o_t_* (*H_t_*,*E_x_*)). The update, denoted Δ*H_t_*, modifies the discriminator output to *p*(*a*|*o_t_* (*H_t_* + Δ*H_t_*,*E_x_*)). Starting from Δ *H_t_* =  0, it is iteratively refined to maximize the likelihood of property *a*:

$$\Delta H_{t}\leftarrow \Delta H_{t}+\alpha \frac{\nabla_{\Delta H_{t}}\log p\left(a|o_{t}\left(H_{t}+\Delta H_{t},E_{x}\right)\right)}{∥\nabla_{\Delta H_{t}}\log p\left(a|o_{t}\left(H_{t}+\Delta H_{t},E_{x}\right)\right){∥}^{\gamma }}$$

where α is the step size and γ is a normalization coefficient. This update is repeated for a tunable number of iterations.

DGIF can further incorporate multiple property predictors to enable multi‐objective optimization. The discriminator can integrate a set of predictors, *p*(*a_i_* |*o_t_* (*H_t_* + Δ*H_t_*,*E_x_*)), each associated with a weighting coefficient β_*i*_, to jointly update *H_t_*:

$$\Delta H_{t}\leftarrow \Delta H_{t}+\alpha \frac{\nabla_{\Delta H_{t}}\sum_{i}\beta_{i}\log p\left(a_{i}|o_{t}\left(H_{t}+\Delta H_{t},E_{x}\right)\right)}{∥\nabla_{\Delta H_{t}}\sum_{i}\beta_{i}\log p\left(a_{i}|o_{t}\left(H_{t}+\Delta H_{t},E_{x}\right)\right){∥}^{\gamma }}$$

$$\;{\tilde{H}}_{t}=H_{t}+\Delta H_{t}$$

By tuning the weighting coefficients β_*i*_, the relative influence of each property can be flexibly adjusted to balance trade‐offs between competing objectives.

The updated history state ${\tilde{H}}_{t}=H_{t}+\Delta H_{t}$ is then reused for forward propagation:

$${\tilde{o}}_{t+1},\;H_{t+1}=Decoder\left(y_{t},\;{\tilde{H}}_{t},E_{x}\right)$$

$${\tilde{y}}_{t+1}∼\;\;{\tilde{p}}_{t+1}=Softmax\left(W{\tilde{o}}_{t+1}\right)$$

allowing the next residue ${\tilde{y}}_{t+1}$ to be sampled under the guidance of the discriminator.

To preserve the foldability of the generated sequences, DGIF introduces a KL divergence regularization term:

$$\;L_{K}=D_{\mathrm{KL}}\;\left({\tilde{p}}_{t+1}∥p_{t+1}\right)=\sum_{x}{\tilde{p}}_{t+1}\left(x\right)\;\log\frac{{\tilde{p}}_{t+1}\left(x\right)}{p_{t+1}\left(x\right)}$$

This constraint prevents $\;{\tilde{p}}_{t+1}$ from deviating excessively from the original distribution  *p*_*t* + 1_, thereby retaining the pretrained model's sequence design capability. Both the discriminator loss and the KL loss contribute jointly to the update of Δ*H_t_*.

To improve computational efficiency, DGIF applies a spatial truncation strategy in which only KV‐cache entries corresponding to residues within 10 Å of the Cα atom of the current residue are updated. The 10 Å cutoff was chosen to cover the local structural environment around the target residue, including direct contacts and interactions with nearby residues [65]. Local neighborhoods of a similar spatial scale are commonly used in structure‐aware protein modeling to capture local residue coupling while reducing the influence of less relevant long‐range information [66].

The weighting coefficients β_*i*_ for different properties were determined through an empirical search on the validation set. Specifically, we generated candidate sequences under different combinations of weighting coefficients and evaluated the resulting changes in thermostability, solubility, and the log‐likelihood assigned by the base inverse folding model. The final weighting combination was selected to maximize thermostability improvement while maintaining the predicted solubility above the wild‐type baseline and avoiding a marked decrease in the base‐model log‐likelihood. This weighting‐selection procedure was used to balance the relative contributions of different property objectives during gradient‐based updates. It also helped mitigate the different convergence rates of the thermostability and solubility losses under identical update settings (Table S2).

To assess the computational overhead introduced by discriminator guidance, we benchmarked the generation speed of DGIF against unguided ESM‐IF1. Using proteins from the S669 benchmark as test cases, unguided ESM‐IF1 generated sequences at 372 ± 16 aa/s on a single NVIDIA RTX A6000 GPU. With discriminator‐guided gradient updates, the generation speed decreased to 69 ± 7 aa/s. This additional computational cost mainly arose from the backpropagation required for discriminator guidance at each generation step.

### 4.3 Thermostability Predictor

The thermostability predictor was trained on the Megascale dataset [34], which comprises over 776 298 substitution mutations with experimentally measured ΔΔG values. To prevent data leakage, clustering was performed on the wild‐type sequences, and all mutation data were assigned to the corresponding clusters. The dataset was then split into training, validation, and test sets in a 7:2:1 ratio. For data augmentation, each mutation was paired with its reverse mutation, assigned a value of −ΔΔG, and both were included as independent samples.

The predictor is a two‐layer multilayer perceptron (MLP) that receives the representations of the mutant and wild‐type sequences from the penultimate layer of ESM‐IF1 as input and outputs the predicted ΔΔG. Specifically, the mutant and wild type are encoded to yield representations $o_{:n}^{mutation}$ and $o_{:n}^{native}$, where n denotes the sequence length. These representations are mean‐pooled independently, and their difference is then passed to the MLP, which produces the predicted ΔΔG.

The model was trained with the mean squared error (MSE) loss and optimized using AdamW [67], with a learning rate of 0.0001. The optimal number of training epochs was selected based on the average Spearman correlation [68] between predicted and experimental ΔΔG values on the validation set. Afterward, the predictor was retrained on the full dataset. All models were trained on a single NVIDIA RTX A6000 GPU.

### 4.4 Solubility Predictor

The solubility discriminator was trained on the dataset curated by Khurana et al. [51], comprising 28 972 soluble and 40 448 insoluble protein sequences, split into training and validation sets at a 9:1 ratio. An independent test set from Chang et al. [50], containing 1000 soluble and 1001 insoluble sequences, was used for evaluation. Protein structures for all sequences were predicted with ESM‐Fold [69].

The predictor is a binary classifier implemented as a two‐layer multilayer perceptron (MLP). Its input is the mean‐pooled protein representation $o_{:n}^{protein}$ derived from ESM‐IF1, where n is the sequence length. The MLP output is passed through a softmax layer to predict solubility. Training was performed with cross‐entropy loss using AdamW with a learning rate of 0.0001.

### 4.5 Molecular Dynamics Simulations of Xylanase and Designed Variants

The native structure of xylanase was obtained from PDB entry 3WUE, while the structures of proteins designed by DG‐Thermo and ESM‐IF1 were predicted using AlphaFold3 [70]. Each initial conformation was centered in a cubic box of 8 nm per side and solvated with water molecules. Sodium and chloride ions were added to neutralize the system and approximate physiological conditions (∼150 mm NaCl). The systems were first subjected to 50 000 steps of energy minimization, followed by equilibration under the NVT ensemble (300 K) and the NPT ensemble (300 K, 1 atm) for 10 ns each. Production molecular dynamics (MD) simulations were then conducted in the NPT ensemble for 100 ns at 450 K.

All MD simulations were carried out using the GROMACS software package [71] (version 2018.x or newer) with the CHARMM36m force field [72]. Temperature and pressure were controlled by the velocity‐rescaling thermostat [73] and the Berendsen barostat [74], respectively. Periodic boundary conditions were applied in all directions [75]. Long‐range electrostatic interactions were calculated using the particle mesh Ewald method [76], and the Verlet cut‐off scheme was employed for neighbor searching with a cutoff of 1.2 nm for both electrostatics and van der Waals interactions. Covalent bonds involving hydrogen atoms were constrained with the LINCS algorithm [77], and a 2 fs integration time step was used.

We further selected ten additional protein systems for supplementary validation, which are randomly sampled from the CATH4.3 dataset. Each system was subjected to a 50‐ns molecular dynamics (MD) simulation at 400 K (Figure S2). The simulation settings were identical to those used for the xylanase system.

### 4.6 Retrospective Analysis of Thermostability‐Directed Evolution Cases

For the retrospective analysis, we selected two reported thermostability‐directed evolution cases: subtilisin E (PDB 1SCJ) and Thermus maltogenic amylase (PDB 1SMA) (Table S3A,B). For each system, wild‐type and evolved variant sequences were constructed according to the mutation combinations reported in the original studies, and the corresponding backbone structure was used as the conditioning input for inverse folding. For each mutation retained in the reported evolutionary trajectory, all 19 non‐wild‐type amino acid substitutions at the same site were enumerated. We then calculated the backbone‐conditioned sequence log‐likelihoods assigned by DG‐Thermo and ESM‐IF1 to each single‐mutant sequence and ranked the 19 substitutions at each site; a smaller rank indicates stronger model support (Table S6A,B). For the multi‐mutant analysis, we calculated the log‐likelihoods of the reported evolved variants and the corresponding wild‐type sequences. The log‐likelihood change relative to the wild type was defined as Δll. To assess whether the scores of combinatorial variants could be explained by independent single‐mutation effects, we compared the observed Δll of each multi‐mutant variant with the sum of Δll values of its component single mutations (Tables S4A,B and S5A,B).

### 4.7 Inverse Folding–Based Identification of Key Mutations

We adopted a strategy inspired by Fei et al. [78], to identify mutations that may enhance both protein thermostability and solubility. The backbone structure of the target protein was provided to DG‐Dual, generating 10 000 structurally compatible sequences. For each model, we identified the most frequently occurring amino acid substitution at each residue position *i* using the following procedure.

For each residue position *i*, the occurrence frequency of an amino acid *x* was defined as:

$$f_{x}\;\left(i\right)=\frac{1}{M}\;\sum_{j=1}^{M}1\left\{x_{i}=x\right\}$$

where M is the number of sampled sequences, and 1{·} is the indicator function, which equals 1 if the condition is satisfied and 0 otherwise. From this, we obtained the appearance frequency of the wild‐type amino acid at position *i*, denoted *f_wt_* (*i*), and the frequencies of all alternative amino acids, denoted *f_mut_* (*i*). The highest mutation frequency at position *i* was then defined as:

$$f_{max}\;\left(i\right)=\max\left\{\;f_{mut}\left(i\right)|f_{mut}\left(i\right)>f_{wt}\left(i\right)\right\}$$

with the corresponding amino acid denoted as $x_{i}^{\mathrm{wut}}$.

To refine the candidate set, two threshold parameters were introduced: a β‐score for global screening and a γ‐score for residues in flexible regions. Residues with *f_max_* (*i*) >  β were retained, while residues located in flexible regions were required to satisfy *f_max_* (*i*) >  γ. Flexible regions were determined using the DSSP algorithm [79], where structural categories such as bends (S) and turns (T) were classified as flexible. The final mutation set was obtained by combining residues identified through both global and flexible‐region screening. From the generated candidate mutations, we randomly selected ten for experimental validation to evaluate the ability of DG‐Dual to design protein variants with simultaneous improvements in thermostability and solubility.

### 4.8 Expression of Rhodococcus Ruber Alcohol Dehydrogenase (RrADH) and the Designed Variants

The wild‐type and the designed mutants of Rhodococcus ruber alcohol dehydrogenase (RrADH) were recombinantly expressed by *Escherichia coli* (*E. coli*). For each protein, a polyhistidine tag was added to its C‐terminus. After codon optimization, the gene sequences were cloned into the pET‐30a vector, and the recombinant plasmids were transformed into *E. coli* BL21 (DE3) strain. The plasmid construction was performed by Shanghai Generay Biotech Co., Ltd. The *E. coli* bacterial cells were cultured in the LB medium (10 g/L tryptone, 5 g/L yeast extract, and 10 g/L NaCl) at 37 °C with shaking at 220 rpm. Cell growth was estimated by the spectrophotometric measurement of the absorption at 600 nm. When the optical density (OD_600_) reached 0.8–1.2, protein expression was induced by adding 1 m isopropyl‐b‐D‐thiogalactoside (IPTG) (#10902ES60, Yeasen, China) with 1:1000 dilution, followed by incubation at 25 °C for 6 h. Then, the cells were harvested by centrifugation (6000 × g, 5 min, 4 °C).

### 4.9 Measurement of Protein Solubility

The harvested *E. coli* cells were weighed and resuspended in PBS buffer (1:20, w/v), which was then disrupted by sonication with 20 kHz at 4 °C. A total of 20 cycles of ultrasonication were performed, and for each cycle, the cells were crushed for 4 s followed by cooling for 6 s. Then, the supernatant was collected by centrifugation (5000 × g, 10 min, 4 °C). Soluble protein expression in the supernatant was quantified by enzymelinked immunosorbent assay (ELISA) using the anti‐histidine antibody. ELISA measurement was carried out after a 10‐fold dilution, and the level of soluble protein in each sample was determined by the value of OD_450/630 nm_.

### 4.10 Determination of Melting Temperature (Tm)

The expressed wild‐type RrADH and the designed variants were purified by Ni‐affinity chromatography using the Ni‐IDA Purose 6 FF column (#A40902‐05, Qianchun Biotechnology, Jiaxing, China). The melting temperature (Tm) of the purified proteins was determined to evaluate the thermal stabilities using the UNcle system (Unchained Labs, USA) based on intrinsic fluorescence. For each protein, 9 µL sample was loaded into the Uni and analyzed in technical duplicates under the linear temperature ramp mode (starting temperature: 25 °C; equilibration time: 180 s; heating rate: 0.4 °C/min; end temperature: 85 °C). The melting temperature (Tm) was determined as the inflection point of the fluorescence intensity curve using the UNcle analysis software.

## Author Contributions

Y.C. and J.L. conceptualized and designed the project. C.L. and Y.C. wrote the code and performed the data analysis. Z.M.L., X.Z., and H.Z. designed and carried out the experimental validation. Y.C. and J.L. wrote the manuscript. All authors discussed and revised the manuscript.

## Conflicts of Interest

The authors declare no conflicts of interest.

## Supporting information




**Supporting File**: advs75988‐sup‐0001‐SuppMat.docx.

## Data Availability

All relevant data supporting the key findings of this study are available within the article and its Supplementary Information files. Datasets used for training and testing the predictors and for assessing DGIF performance are available in the cited references. The code for DGIF and its implementation based on ESM‐IF1 can be found at https://github.com/aweqardf/ESM‐IF1‐DG.
