# Supplementary material for: Discriminator‐Guided Inverse Folding for Multi‐Property Protein Design
Source: Adv Sci (Weinh). 2026 Jun 9:e75988. Online ahead of print. doi: 10.1002/advs.75988 (PMC13336499; doi:10.1002/advs.75988)
Supplement: Supplementary file 1 — Supporting File: advs75988‐sup‐0001‐SuppMat.docx. [file ADVS-9999-e75988-s001.docx]

**Discriminator-Guided Inverse Folding for Multi-Property Protein Design**

Yuchuan Zheng^1†^, Chuyi Liu^2†^, Zhao Ming Liu^3,4†^, Mao Su^5^, Chenyu Tang^6^, Xiang Zheng^3,4^, Hao Zhang^3,4^, Jingyuan Li^1,2,*^

^1^Institute for Advanced Study in Physics, Zhejiang University, Hangzhou 310058, China

^2^School of Physics, Zhejiang University, Hangzhou 310058, China

^3^The Sixth Laboratory, National Vaccine and Serum Institute (NVSI), Beijing 101111, China.

^4^National Engineering Research Center for Novel Vaccines, Beijing 101111, China.

^5^Shanghai Artificial Intelligence Laboratory, Shanghai, China.

^6^ Laboratoire International Associ´e Centre National de la Recherche Scientifique et University of Illinois at Urbana-Champaign, Unit´e Mixte de Recherche n◦7019,

Universit´e de Lorraine, B.P. 70239, 54506 Vandœuvre-l`es-Nancy cedex, France

^†^These authors contributed equally to this work.

*Corresponding Authors: Jingyuan Li ([jingyuanli@zju.edu.cn](mailto:jingyuanli@zju.edu.cn)).

**Supplementary Figures**


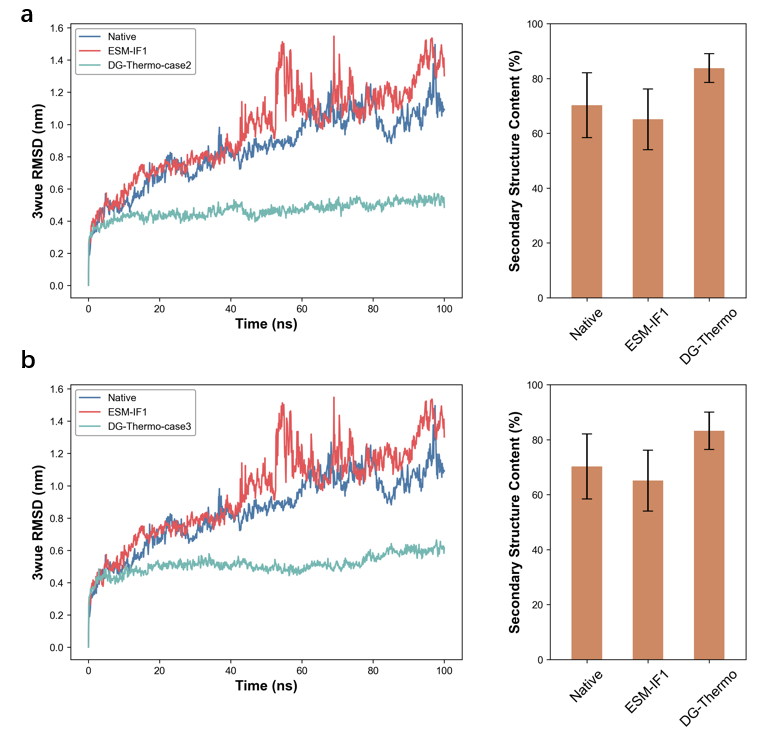


**Fig. S1 | Molecular dynamics (MD) evaluation of the thermal stability of xylanase variants designed by DG-Thermo.** **a–b,** Root Mean Square Deviation (RMSD) trajectories and average secondary structure contents over 100-ns simulations at 450K for DG-Thermo-designed variants, compared with the wild-type protein and variants designed by ESM-IF1.


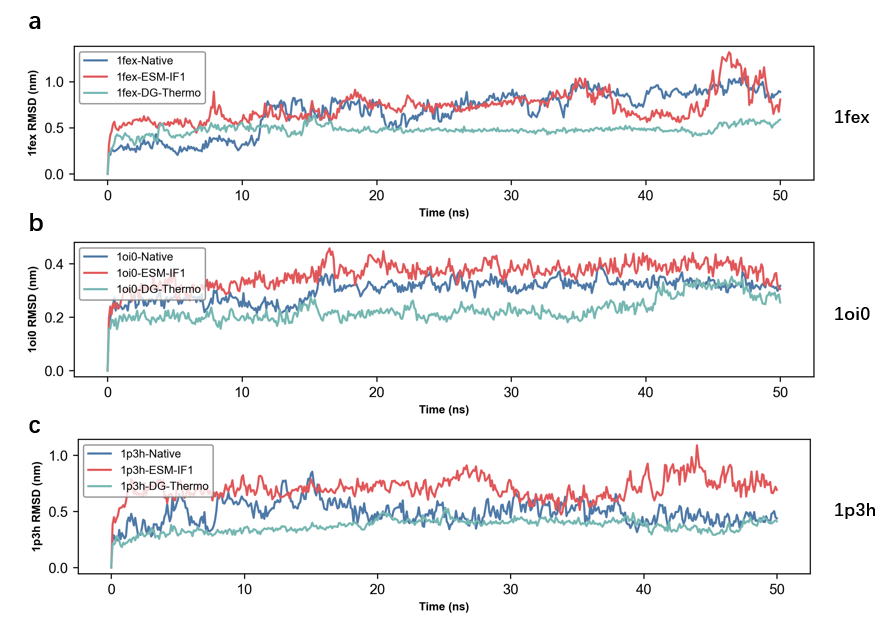


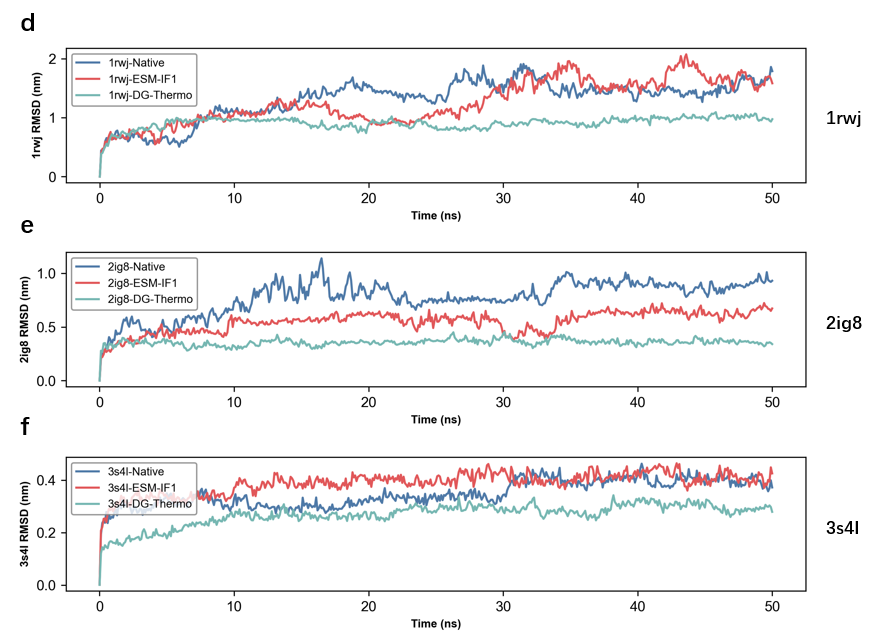


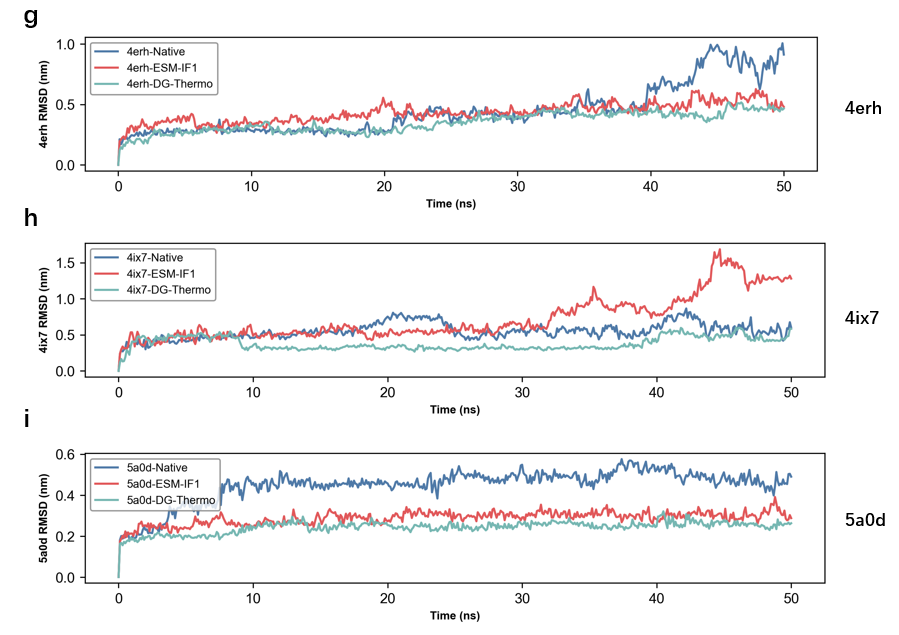

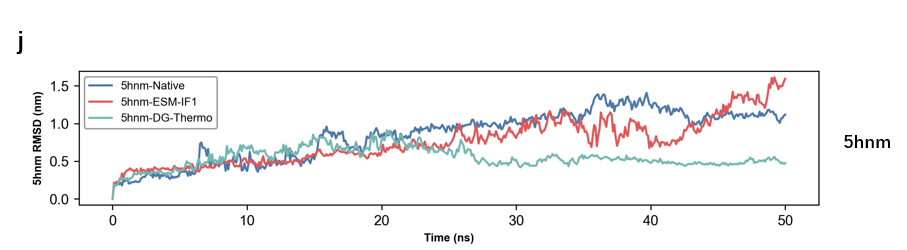


**Fig. S2 | MD simulation validations of the thermal stability in additional protein systems from CATH4.3 dataset**^1^**.** Wild-type proteins and variants redesigned by DG-Thermo and ESM-IF1 were subjected to 50-ns MD simulations at 400 K. **a–j,** RMSD trajectories over time for the wild-type, ESM-IF1-designed variants, and DG-Thermo-designed variants of each system.


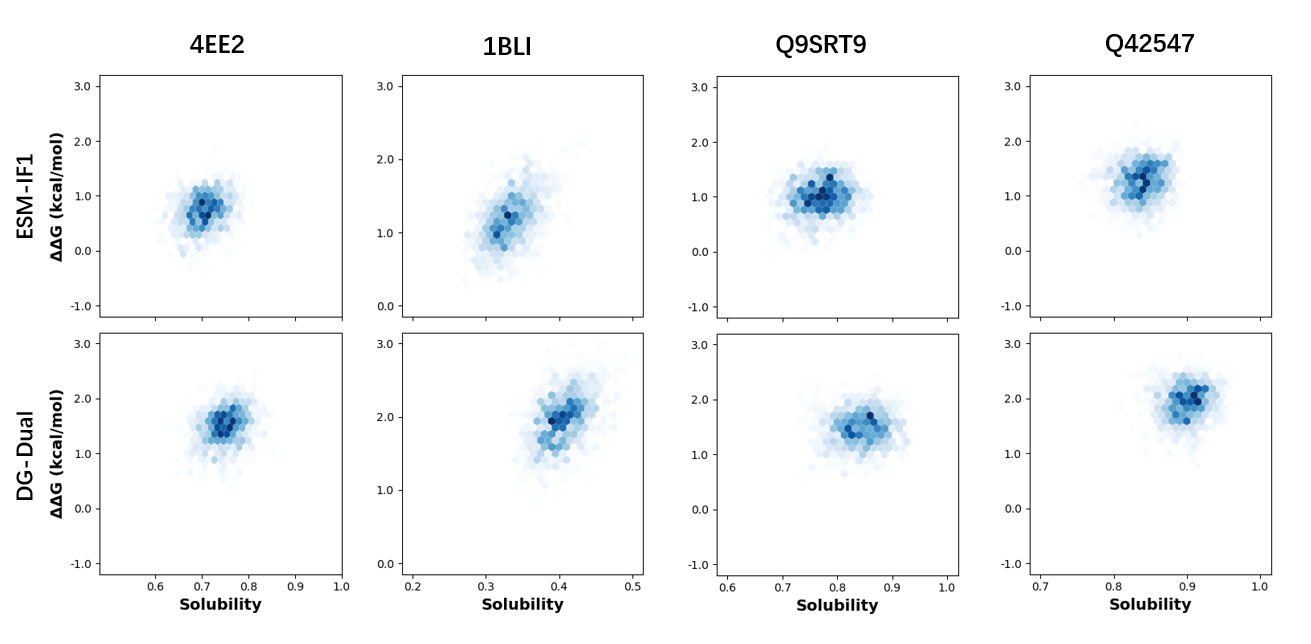


**Fig. S3 |** **Predicted ΔΔG values and solubility scores for protein variants redesigned by DG-Dual and ESM-IF1.** Variants designed by ESM-IF1 are shown above, and those by DG-Dual below. The corresponding PDB IDs of the protein backbones are indicated at the top.


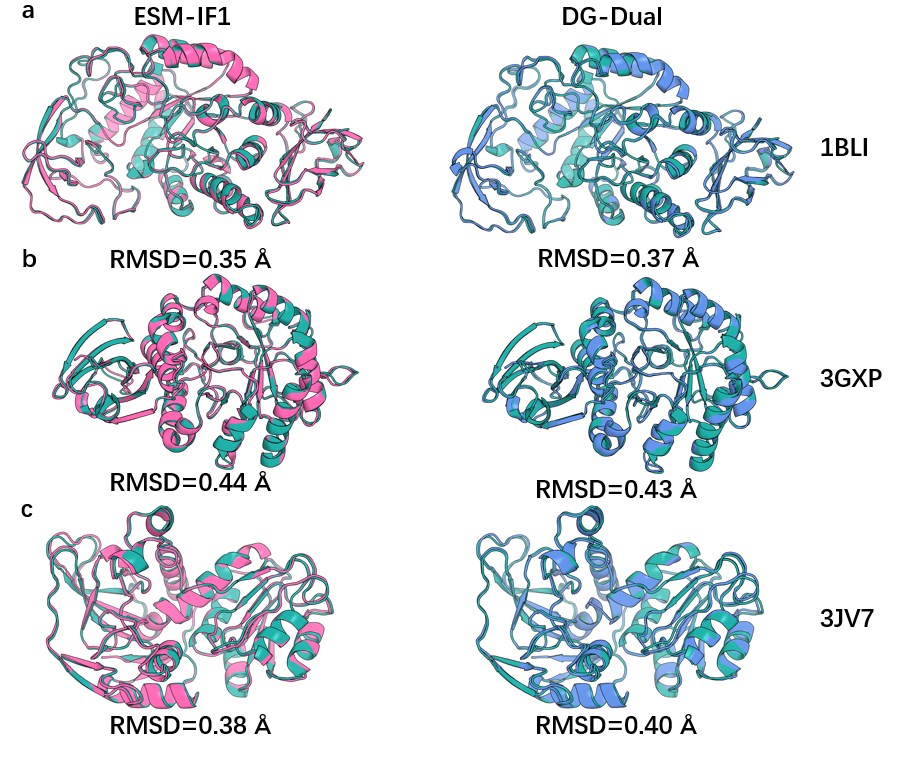


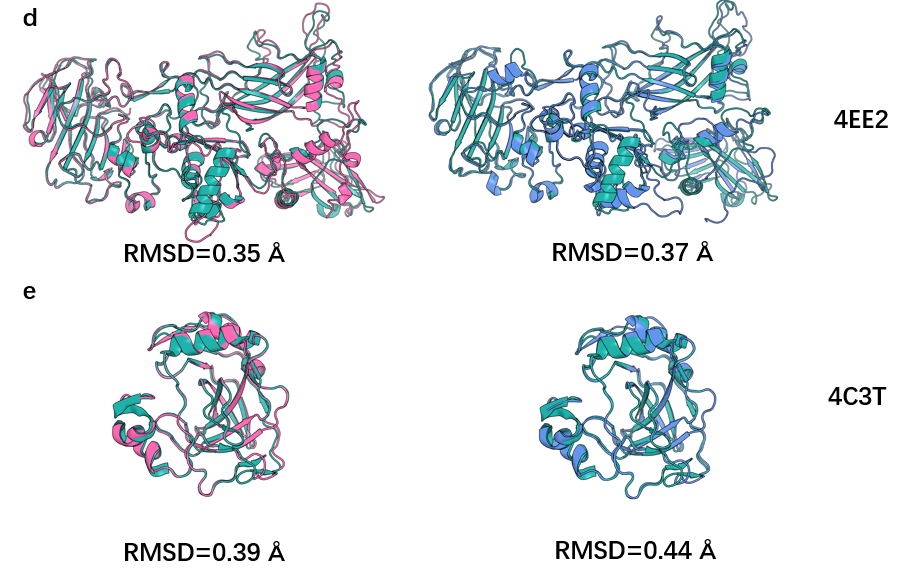


**Fig. S4 | RMSD comparisons between AlphaFold3**^2^**-predicted structures of DG-Dual or ESM-IF1 designed proteins and their native counterparts.**


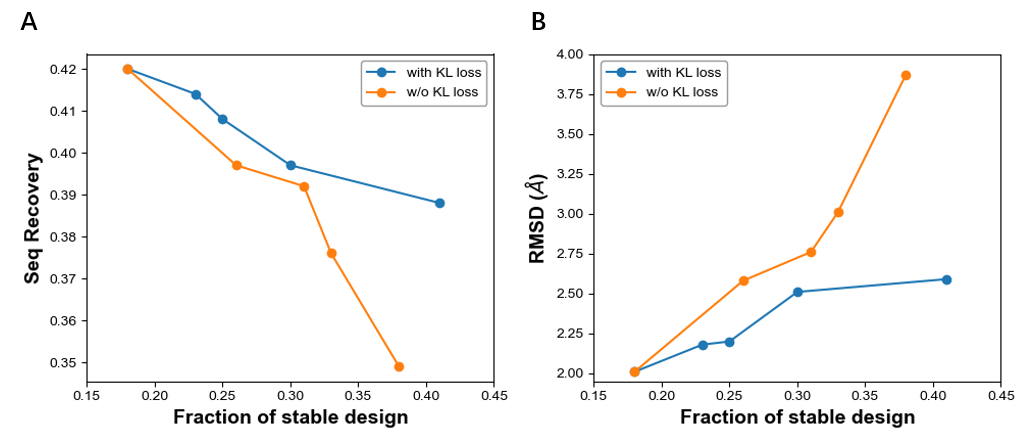


**Fig. S5 | Relationship between property optimization and foldability of designed proteins. a,** Correlation between the success rate of designing proteins with enhanced thermostability and sequence recovery. **b,** Correlation between the success rate and the root-mean-square deviation (RMSD) between designed proteins and their native counterparts. Each data point was derived from statistical results on 20 different proteins, with 100 designed sequences generated for each protein.


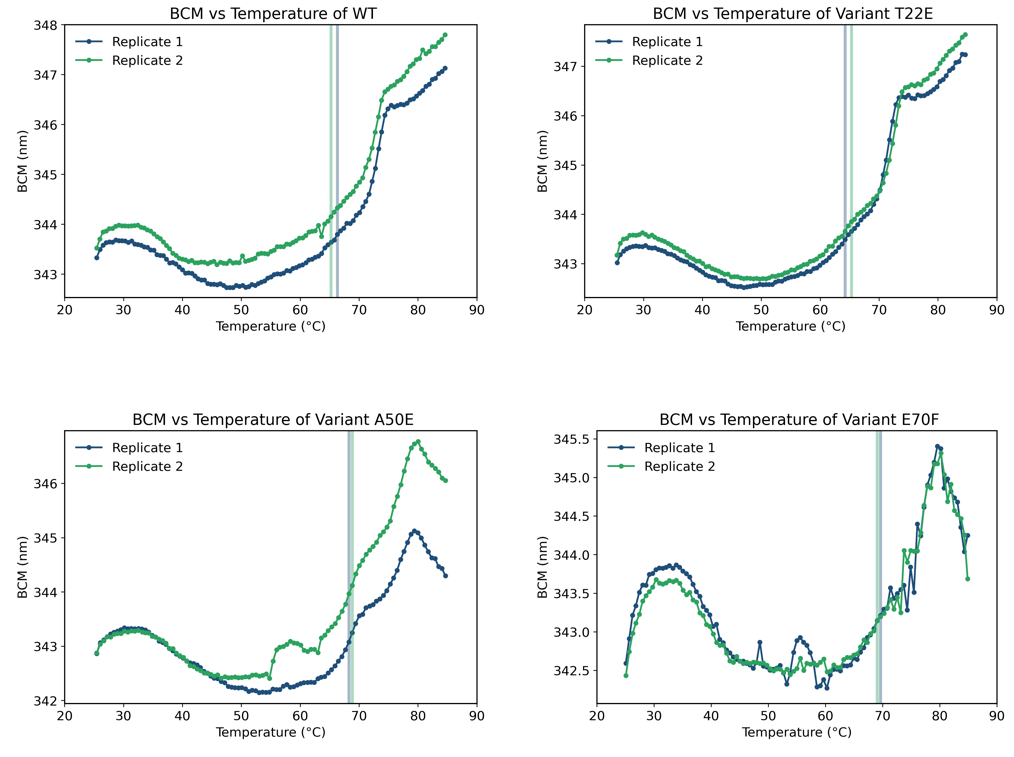


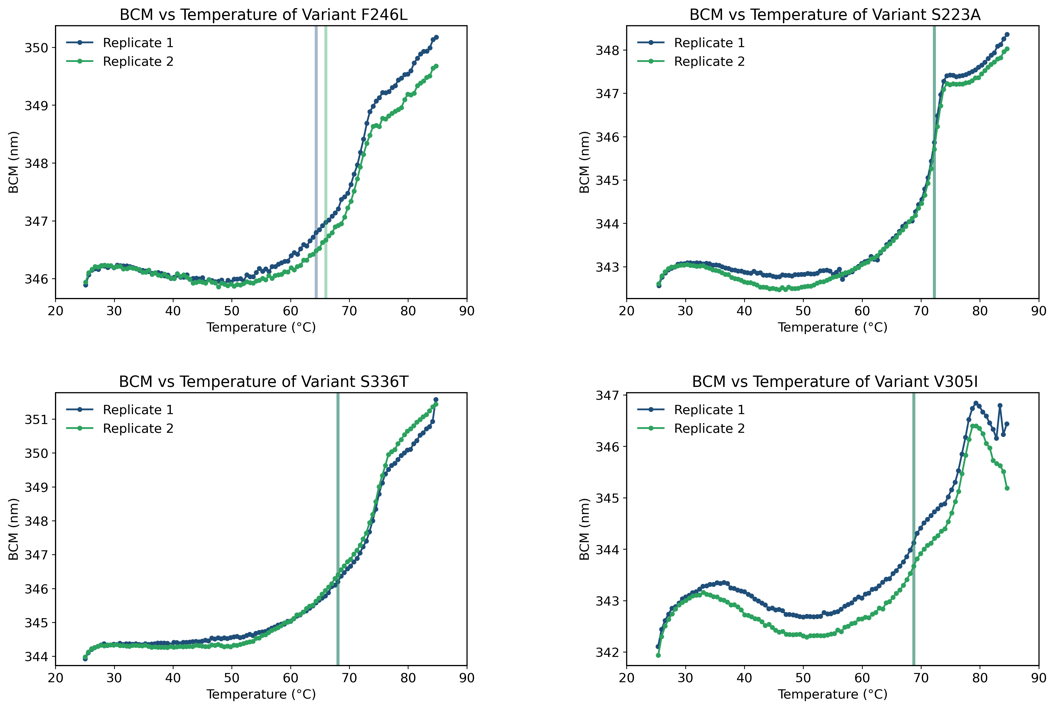


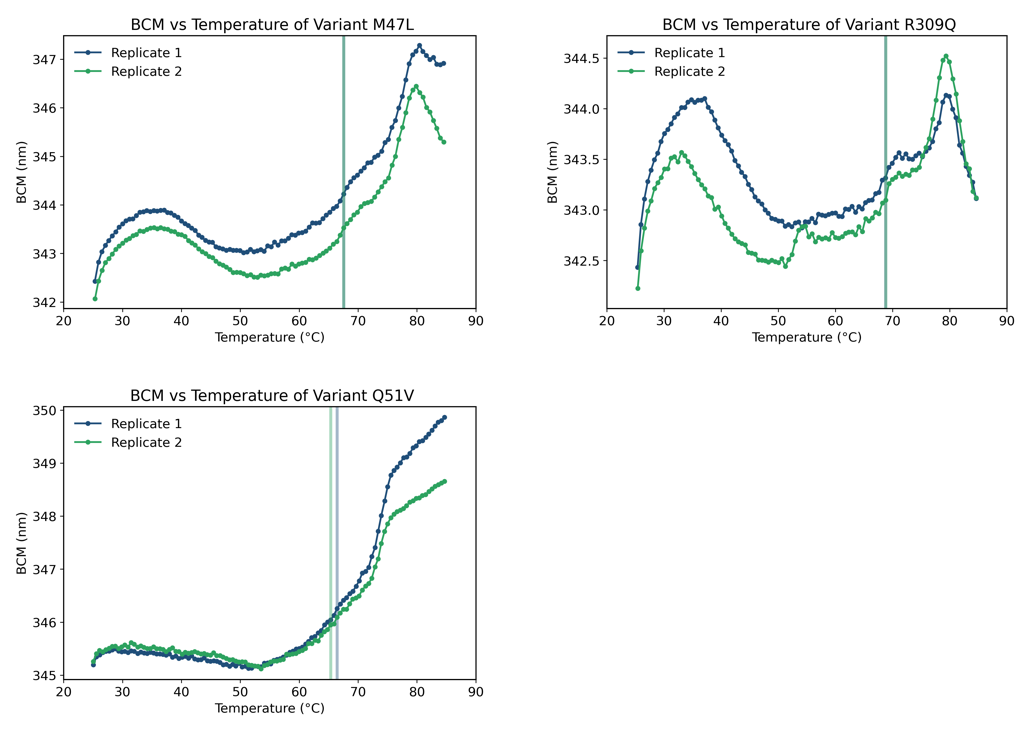


**Fig. S6 | Temperature-dependent barycentric mean (BCM) curves of the wild-type and mutant RrADH proteins measured using the UNcle system.** Each sample was measured in duplicate, and the corresponding Tm values are shown as vertical lines in the respective colors.


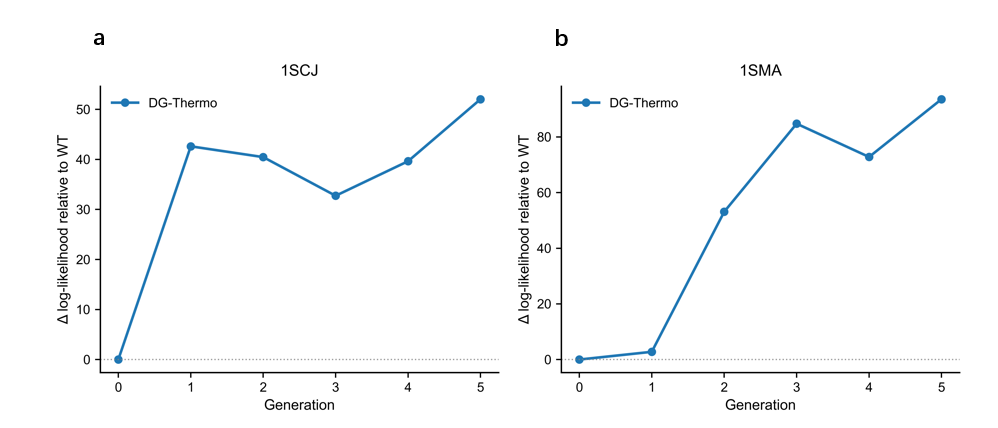


**Fig. S7 | DG-Thermo log-likelihoods along reported directed-evolution trajectories**^3,4^**.** a, Change in log-likelihood relative to the wild type for the reported variants along the directed-evolution trajectory of subtilisin E (PDB 1SCJ). b, Change in log-likelihood relative to the wild type for the reported variants along the directed-evolution trajectory of Thermus maltogenic amylase (PDB 1SMA). In both systems, DG-Thermo assigns higher likelihoods to the final successful variants than to the corresponding wild-type sequences.

**Supplementary Tables**

**Table S1. Solubility of RrADH variants at different dilution ratios, measured by ELISA (OD₄₅₀/₆₃₀ nm).** The results obtained at a 10× dilution were used for quantitative analysis.

| **Variant** | **2× Dilution** | **10× Dilution** | **100× Dilution** | **1000× Dilution** |
| --- | --- | --- | --- | --- |
| WT | **1.379** | **0.767** | **0.890** | **0.581** |
| T22E | 1.758 | **1.626** | 1.280 | 0.992 |
| A50E | 1.877 | **1.616** | 1.456 | 1.177 |
| E70F | 1.971 | **1.511** | 1.405 | 0.773 |
| F246L | 1.850 | **1.433** | 1.346 | 0.991 |
| S223A | 1.567 | **1.337** | 1.187 | 0.666 |
| S336T | 1.420 | **1.299** | 1.120 | 0.438 |
| V305I | 1.765 | **1.226** | 1.196 | 0.745 |
| M47L | 1.506 | **1.207** | 0.991 | 0.710 |
| R309Q | 1.754 | **1.201** | 0.946 | 0.726 |
| Q51V | 1.287 | **1.081** | 1.167 | 0.535 |

**Table S2. Effect of the number of iterations and step size on the convergence behavior of the Discriminator-guided protein sequence generation.** For each target property, the ratio $loss_{t}/loss_{0}$ represents the relative loss after one token generation step normalized by its initial value, reflecting the effective convergence rate under the given hyperparameters. The table shows that under identical hyperparameters, convergence speed varies across different properties, suggesting property-dependent response to gradient-based perturbations.

| **Num_iter** | **Stepsize** | $\boldsymbol{los}\boldsymbol{s}_{\boldsymbol{t}}^{\boldsymbol{solu}}\boldsymbol{/los}\boldsymbol{s}_{\boldsymbol{0}}^{\boldsymbol{solu}}$ | $\boldsymbol{los}\boldsymbol{s}_{\boldsymbol{t}}^{\boldsymbol{thermo}}\boldsymbol{/los}\boldsymbol{s}_{\boldsymbol{0}}^{\boldsymbol{thermo}}$ |
| --- | --- | --- | --- |
| 2 | 0.1 | 0.894 | 0.765 |
| 2 | 0.3 | 0.798 | 0.955 |
| 3 | 0.1 | 0.589 | 0.637 |
| 3 | 0.3 | 0.780 | 0.859 |

**Table S3A.** Experimentally reported directed-evolution trajectory and thermal-stability measurements for the 1SCJ case study used in the retrospective analysis. T50 is reported as described in the original literature^3^.

| **Generation** | **Variant** | **Mutations present** | **Total mutations** | **T50 (°C)** |
| --- | --- | --- | --- | --- |
| 0 | WT | - | 0 | 59.2 |
| 1 | 4A5 | N181D N218S | 2 | 67.4 |
| 2 | 45B7 | G166R N181D S194P N218S | 4 | 72.3 |
| 3 | 16D11 | G166R N181D S194P N218S P14L | 5 | 73.3 |
| 4 | 8B3 | P14L N76D G166R N181D S194P N218S | 6 | 74.4 |
| 5 | 3H5 | N118S S161C P14L N76D G166R N181D S194P N218S | 8 | 76.4 |

**Table S3B.** Experimentally reported directed-evolution trajectory and thermal-stability measurements for the 1SMA case study used in the retrospective analysis. Tm values are reported as described in the original literature^4^.

| **Generation** | | **Variant** | **Mutations present** | **Total mutations** | **Tm (°C)** |
| --- | --- | --- | --- | --- | --- |
| 0 | WT | - | 0 | 76.2 |  |
| 1 | 1B100 | A398V | 1 | 77.2 |  |
| 2 | 2A39 | S169N A398V P453L | 3 | 78.7 |  |
| 3 | 3C71 | S169N A398V P453L I333V | 4 | 79.1 |  |
| 4 | 4B78 | S169N A398V P453L I333V R26Q | 5 | 79.4 |  |
| 5 | DM | S169N A398V P453L I333V R26Q Q411L M375T | 7 | 86.9 |  |

**Table S4A.** DG-Thermo and ESM-IF1 log-likelihoods for the single mutations involved in the directed-evolution trajectory of 1SCJ. Log-likelihoods and DG-Thermo likelihood changes relative to the wild type are reported to three decimal places. ESM-IF1 likelihood changes relative to the wild type are reported in scientific notation because they are close to zero.

| **Single mutation** | **ll_DG-Thermo** | **ll_ESM-IF1** | **Δ ll_DG-Thermo relative to WT** | **Δ ll_ESM-IF1 relative to WT** |
| --- | --- | --- | --- | --- |
| N181D | -327.783 | -200.627 | 17.565 | 6.0 × 10⁻⁶ |
| N218S | -328.716 | -200.627 | 16.633 | -2.0 × 10⁻⁵ |
| G166R | -311.198 | -200.627 | 34.152 | 3.0 × 10⁻⁶ |
| S194P | -321.315 | -200.627 | 24.034 | -1.7 × 10⁻⁵ |
| P14L | -317.976 | -200.627 | 27.372 | -1.1 × 10⁻⁵ |
| N76D | -310.449 | -200.627 | 34.900 | -1.9 × 10⁻⁵ |
| N118S | -336.313 | -200.627 | 9.035 | -2.0 × 10⁻⁶ |
| S161C | -323.316 | -200.627 | 22.032 | -3.2 × 10⁻⁵ |

**Table S4B.** DG-Thermo and ESM-IF1 log-likelihoods for the single mutations involved in the directed-evolution trajectory of 1SMA. Log-likelihoods and DG-Thermo likelihood changes relative to the wild type are reported to three decimal places. ESM-IF1 likelihood changes relative to the wild type are reported in scientific notation because they are close to zero.

| **Single mutation** | **ll_DG-Thermo** | **ll_ESM-IF1** | **Δ ll_DG-Thermo relative to WT** | **Δ ll_ESM-IF1 relative to WT** |
| --- | --- | --- | --- | --- |
| A398V | -1287.867 | -814.217 | 2.777 | -3.4 × 10⁻⁵ |
| S169N | -1272.713 | -814.217 | 17.931 | 0.0 |
| P453L | -1284.583 | -814.217 | 6.061 | 3.7 × 10⁻⁵ |
| I333V | -1278.274 | -814.217 | 12.370 | -1.0 × 10⁻⁶ |
| R26Q | -1275.442 | -814.217 | 15.202 | 4.2 × 10⁻⁵ |
| Q411L | -1284.114 | -814.217 | 6.530 | -2.5 × 10⁻⁵ |
| M375T | -1283.255 | -814.217 | 7.389 | 4.5 × 10⁻⁵ |

**Table S5A.** DG-Thermo and ESM-IF1 log-likelihoods for the reported variants along the directed-evolution trajectory of 1SCJ. Variant names follow the literature reports; mutation lists are shown exactly as used in the model calculations. Log-likelihoods and DG-Thermo likelihood changes relative to the wild type are reported to three decimal places. ESM-IF1 likelihood changes relative to the wild type are reported in scientific notation because they are close to zero.

| **Generation** | **Variant** | **Mutations** | **ll_DG-Thermo** | **ll_ESM-IF1** | **Δll_DG-Thermo relative to WT** | **Δll_ESM-IF1 relative to WT** |
| --- | --- | --- | --- | --- | --- | --- |
| 0 | WT | - | -345.349 | -200.627 | 0.000 | 0.0 |
| 1 | 4A5 | N181D, N218S | -302.738 | -200.627 | 42.610 | -3.0 × 10⁻⁶ |
| 2 | 45B7 | G166R, N181D, S194P, N218S | -304.890 | -200.627 | 40.458 | -3.6 × 10⁻⁵ |
| 3 | 16D11 | P14L, G166R, N181D, S194P, N218S | -312.617 | -200.627 | 32.731 | -2.1 × 10⁻⁵ |
| 4 | 8B3 | P14L, N76D, G166R, N181D, S194P, N218S | -305.713 | -200.627 | 39.635 | -4.0 × 10⁻⁶ |
| 5 | 3H5 | N118S, S161C, P14L, N76D, G166R, N181D, S194P, N218S | -293.322 | -200.627 | 52.026 | -2.7 × 10⁻⁵ |

**Table S5B.** DG-Thermo and ESM-IF1 log-likelihoods for the reported variants along the directed-evolution trajectory of 1SMA. Variant names follow the literature reports; mutation lists are shown exactly as used in the model calculations. Log-likelihoods and DG-Thermo likelihood changes relative to the wild type are reported to three decimal places. ESM-IF1 likelihood changes relative to the wild type are reported in scientific notation because they are close to zero.

| **Generation** | **Variant** | **Mutations** | **ll_DG-Thermo** | **ll_ESM-IF1** | **Δll_DG-Thermo relative to WT** | **Δll_ESM-IF1 relative to WT** |
| --- | --- | --- | --- | --- | --- | --- |
| 0 | WT | - | -1290.644 | -814.217 | 0.000 | 0.0 |
| 1 | 1B100 | A398V | -1287.867 | -814.217 | 2.777 | -3.4 × 10⁻⁵ |
| 2 | 2A39 | S169N, A398V, P453L | -1237.537 | -814.217 | 53.107 | 4.2 × 10⁻⁵ |
| 3 | 3C71 | S169N, A398V, P453L, I333V | -1205.879 | -814.217 | 84.765 | -4.0 × 10⁻⁵ |
| 4 | 4B78 | S169N, A398V, P453L, I333V, R26Q | -1217.838 | -814.217 | 72.806 | 6.9 × 10⁻⁵ |
| 5 | DM | S169N, A398V, P453L, I333V, R26Q, Q411L, M375T | -1197.084 | -814.217 | 93.560 | -4.0 × 10⁻⁶ |

**Table S6A.** Site-wise ranks of experimentally retained beneficial single mutations in the 1SCJ directed-evolution case study. Ranks were recalculated after excluding the wild-type residue, so that each mutation is ranked among the 19 non-wild-type amino acid substitutions at the same site. Lower rank indicates better prioritization.

| Single mutation | Rank in ESM-IF1 (excluding WT) | Rank in DG-Thermo (excluding WT) |
| --- | --- | --- |
| P14L | 1 | 1 |
| N76D | 1 | 1 |
| N118S | 1 | 1 |
| S161C | 15 | 9 |
| G166R | 16 | 14 |
| N181D | 1 | 1 |
| S194P | 8 | 6 |
| N218S | 1 | 1 |

**Table S6B.** Site-wise ranks of experimentally retained beneficial single mutations in the 1SMA directed-evolution case study. Ranks were recalculated after excluding the wild-type residue, so that each mutation is ranked among the 19 non-wild-type amino acid substitutions at the same site. Lower rank indicates better prioritization.

| Single mutation | Rank in ESM-IF1 (excluding WT) | Rank in DG-Thermo (excluding WT) |
| --- | --- | --- |
| R26Q | 4 | 1 |
| S169N | 17 | 15 |
| I333V | 1 | 1 |
| M375T | 1 | 1 |
| A398V | 9 | 6 |
| Q411L | 14 | 5 |
| P453L | 8 | 6 |

**Table S7.** Performance comparison between DG-Thermo and other thermostability prediction baselines on the S669 benchmark. ESM-IF1 (DPO) denotes the single-property fine-tuned baseline trained using direct preference optimization on the Megascale dataset. The ThermoMPNN result is taken from the published literature^5^.

| **Model** | **Pearson correlation on S669** |
| --- | --- |
| ESM-IF1 | 0.297 |
| ESM-IF1 (DPO) | 0.466 |
| ThermoMPNN | 0.430 |
| DG-Thermo | **0.491** |

**Reference**

1. Singh, A. Predicted protein structures expand the CATH database. *Nat. Methods* **20**, 483–483 (2023).

2. Abramson, J. *et al.* Accurate structure prediction of biomolecular interactions with AlphaFold 3. *Nature* **630**, 493–500 (2024).

3. Zhao, H. & Arnold, F. H. Directed evolution converts subtilisin E into a functional equivalent of thermitase. *Protein Eng. Des. Sel.* **12**, 47–53 (1999).

4. Kim, Y.-W. *et al.* Directed Evolution of Thermus Maltogenic Amylase toward Enhanced Thermal Resistance. *Appl. Environ. Microbiol.* **69**, 4866–4874 (2003).

5. Dieckhaus, H., Brocidiacono, M., Randolph, N. Z. & Kuhlman, B. Transfer learning to leverage larger datasets for improved prediction of protein stability changes. *Proc. Natl. Acad. Sci.* **121**, e2314853121 (2024).
